# What is the evidence for the impacts of airborne anthropogenic noise on wildlife? A systematic map update

**DOI:** 10.1186/s13750-025-00368-3

**Published:** 2025-07-26

**Authors:** Léa Terray, Benjamin Petiteau, Guillaume Dutilleux, Sylvie Vanpeene, Pamela Amiard, Romain Sordello, Yorick Reyjol

**Affiliations:** 1 PATRINAT (OFB - MNHN), Paris, France; 2 Acoustics Group, Department of Electronic Systems, NTNU - Norwegian University of Science and Technology, Trondheim, Norway; 3 INRAE – Aix Marseille Université, UMR RECOVER, Aix-en-Provence, France

**Keywords:** Noise pollution, Environmental stressor, Acoustic disturbance, Traffic, Urbanization, Behaviour, Reproduction, Physiology, Communication, Diversity

## Abstract

**Background:**

Noise from human activities is a major concern for wildlife, with numerous studies demonstrating significant impacts. In 2020, Sordello and collaborators systematically mapped the literature on the impacts of anthropogenic noise on wildlife up to 2018. Since then, research on this topic has continued to grow steadily. To reflect these developments, we present an updated systematic map encompassing studies published through 2023, exclusively focused on airborne noise.

**Methods:**

The method follows the a priori protocol published by Sordello and collaborators in 2019. The present work includes literature searches by Sordello et al. (2020) and a complementary search update performed on 2020–2023. Literature from Sordello et al. (2020) was re-screened to align with the updated scope, now restricted to airborne noise. For the update, both peer-reviewed and grey literature were retrieved from Scopus, the Web of Science Core Collection, and Google Scholar. Titles, abstracts, and full texts were screened by eligibility criteria, and included articles were coded. We included all wild terrestrial or semi-aquatic taxonomic groups, and anthropogenic noise from various sources (e.g., transport, urban, recreational) was considered, along with all relevant outcomes (e.g., behaviour, reproduction, physiology).

**Review findings:**

Sordello et al. (2020) provided 1,794 articles, of which 466 were retained after re-screening the full texts. The search update yielded 13,698 citations, resulting in 397 relevant articles. A total of 863 articles were included in the map (665 primary research studies, 196 reviews and meta-analyses, 2 modelling papers). Most studies have been conducted in the USA. Birds are the most studied taxonomic group (64%), followed by mammals (22%); transportation is the most studied source of noise (43%), followed by urban noise (24%); behaviour (27%) and vocal communication (25%) are the most studied outcomes.

**Conclusion:**

The map represents an updated state of the art on the impact of airborne anthropogenic noise on wildlife and can serve as a starting point for further syntheses of evidence. Three clusters of knowledge were identified as suitable candidates for future syntheses: (1) What is the impact of anthropogenic noise on mammals’ behaviour? (2) What is the impact of anthropogenic noise on birds’ reproductive success? (3) What is the impact of anthropogenic noise on species richness and diversity? In addition, the knowledge gaps identified may be used to inform future research and address the apparent imbalance in the published research: many taxonomic groups are still understudied (e.g., especially reptiles and arachnids), many potential sources of noise disturbance are neglected (e.g., recreational and military noise) and the impacts of noise are unevenly studied between taxonomic groups.

**Supplementary Information:**

The online version contains supplementary material available at 10.1186/s13750-025-00368-3.

## Background

Sound is a fundamental component of most environments. A large number of species both perceive and produce sounds [1, 2], which are essential for communication among conspecifics and for detecting prey or potential threats. Natural soundscapes typically consist of a mix of meaningful and irrelevant sounds, the latter being referred to as ‘noise.’ Anthropogenic noise is a recent addition to these environments and can interfere with natural acoustic conditions, potentially disrupting wildlife behaviour, health, and habitats. Predominantly originating from urban traffic, industrial and commercial activities, anthropogenic noise can extend far beyond city boundaries, reaching remote and even protected areas such as national parks, where it may arise from ecotourism or aircraft activity. This pervasive presence of anthropogenic noise in natural habitats is commonly referred to as ‘noise pollution’.

Noise pollution is identified as a significant stressor since it involves many detrimental impacts on wildlife, affecting individuals as well as the overall ecosystem’s health through a variety of mechanisms. As a sensory pollutant, noise can lead to auditory masking, distraction, and disorientation [3]. Auditory masking refers to the fact that noise can mask biological signals, implying that an animal may miss prey or fail to detect the arrival of a predator [4, 5]. Distraction can lead to an animal missing a signal by reallocating its attention toward non-relevant acoustic signals. As a result, animals may live in a permanent state of stress due to increasing their vigilance, which can generate physiological reactions [6] or can lead to behavioural changes, causing animals to hide or flee [7]. Both masking and distraction can result in reproductive interference [8] or failure to receive warnings of predators [5], thus affecting fitness. Distraction may result in reduced foraging efficiency or less time allocated to resting, ultimately affecting the overall well-being [9, 10]. Misleading occurs when a sensory pollutant is mistaken for a natural cue, triggering an inappropriate, often maladaptive response. Consequences can result in collisions [11] or predation [12] and lead to increased mortality.

In 2020, Sordello and collaborators [13] systematically mapped the literature on the impacts of anthropogenic noise on wildlife, covering publications up to 2018. This literature base covers all taxonomic groups, all sources of anthropogenic noise, and all possible outcomes, and implements a thorough search strategy to provide a reliable overview of the topic. This systematic map revealed an extended literature on the impacts of anthropogenic noise on wildlife. A very large number of scientific publications related to the topic were found (1794 relevant articles) [13]. Several knowledge clusters were identified regarding some taxonomic groups (mammals, birds, fish) and some noise sources (transportation, industrial, urban).

The Collaboration for Environmental Evidence recommends that environmental reviews should be updated every five years [14, 15]. Moreover, anthropogenic noise is a research hotspot that has received increasing attention over the past years [13]. Preliminary scoping revealed a significant number of new studies and reviews published since Sordello et al. (2020) [13]. An up-to-date systematic map is essential to ensure a comprehensive understanding of the current state of knowledge [15] and to assess whether and how the identified knowledge gaps are being addressed. In addition, we now know that the well-being of humans and non-humans is interconnected, as highlighted by the “One Health” concept developed by the United Nations [16]. In this context, the systematic map provides a valuable scientific, up-to-date, and comprehensive resource for a wide range of stakeholders, including policymakers, companies, and conservation practitioners.

Here, we present an updated systematic map focused on airborne anthropogenic noise (ANN), building on Sordello et al. (2020) [13] – which, to our knowledge, remains the most extensive literature base on anthropogenic noise and wildlife – by extending the coverage to the period 2019–2023. To perform this update, we followed the same protocol published by Sordello et al. [17] in 2019 and we complied with the updated recommendations provided by Pullin [18] and Bayliss et al. [15]. First, we present a synthesis of the overall literature published on the topic until 2023, by updating the literature base from Sordello et al. (2020) [13]. Secondly, the knowledge clusters and gaps are discussed, along with an analysis of how the scientific community’s interest in the topic evolved over time.

## Stakeholders/expert engagement

This systematic map update was initiated following a call for projects launched in 2022 by the French Foundation for Research on Biodiversity (FRB), the Ministry of Ecological Transition (MTE), and the French Biodiversity Office (OFB), entitled “Impacts on terrestrial biodiversity in the Anthropocene”. The project is managed by PatriNat, a joint research unit funded by the French Biodiversity Office (OFB), the National Centre for Scientific Research (CNRS) and the French National Museum of Natural History (MNHN) and the French National Research Institute for Sustainable Development (IRD).

To support the project, we have assembled a group of experts in the fields of noise pollution and eco-acoustic, coming from varied institutions: the French National Museum of Natural History (MNHN), the Institute for Research and Development (IRD), the National Research Institute for Agriculture, Food and the Environment (INRAE), the Centre for Studies and Expertise on Risks, the Environment, Mobility and Urban Planning (CEREMA), the French Agency for Food, Environmental and Occupational Health and Safety (ANSES) and the Norwegian University of Science and Technology (NTNU). The experts have been consulted at various key stages of the project, to ensure its robustness, reliability and consistency. They provided support to the review team in redefining the scope of the systematic map, and defining the eligibility inclusion/exclusion criteria. They also contributed to screening and coding stages.

## Objective of the review

The aim of the systematic map was to provide an up-to-date comprehensive overview of the available knowledge on the impacts of ANN on wildlife. The scope of the map update (e.g. restriction to airborne noise) was fixed with the stakeholders, but is all the more justified as reviews of the literature on underwater anthropogenic noise have recently been published (e.g. [19, 20]).

### Primary question

What evidence exists regarding the impacts of airborne anthropogenic noise on wildlife?

### Secondary question

Which taxonomic groups/noise sources/outcomes have received the most attention? Have these trends varied over time?

### Components of the primary question

To meet the requirements of the call for projects in which it is rooted, the map focuses exclusively on airborne noise. The scope of the updated systematic map is therefore restricted compared to Sordello et al. (2020) [13].

- **Population**: All terrestrial taxonomic groups were included as well as semi-aquatic groups, while strictly aquatic groups were excluded.
- **Exposure**: We considered all ANN propagating in a terrestrial environment, from all sources.
- **Comparators**: We considered all kinds of comparators.
- **Outcomes**: We considered all types of outcomes.

## Methods

This systematic map complies with the Collaboration for Environmental Evidence Guidelines and Standards for Evidence Synthesis in Environmental Management version 5.1 [14], and conforms to ROSES reporting standards for systematic evidence [21] (Additional file 1). The method was based on the systematic map protocol published by Sordello et al. [17] in 2019 (with a few differences all detailed in the following section). The constitution of our literature base was achieved in two stages: (1) the recovery and refined screening of the database from Sordello et al. (2020) [13] according to the new scope, (2) the update of this database through the search, screening and coding of articles published between 2020 and 2023.

### Deviations from the protocol

Sordello et al. (2020) [13] exposed several deviations from their own protocol, which we applied to the literature from the search update for the sake of consistency:

- For each search string performed on Google Scholar, we exported the first 1000 hits instead of the first 300 hits stipulated by the protocol.
- We extracted the bibliography of 51 key reviews published between 2020 and 2023 (in addition to the 37 reviews selected by Sordello et al. (2020) [13]).
- The design comparator (e.g., CE, BAE, BACE) was not extracted.
- Articles were not split into studies when several species, sources of noise or outcomes were mentioned. Therefore, we coded the multiple aspects of these articles on a single line in the map database.

Some deviations occurred in the present map with regard to the restriction of the scope, and were applied to both the refining process of Sordello et al. (2020) [13] and to the updating process:

- Eligibility criteria were revised to reflect the restriction of the scope to airborne noise.
- The variables used for the coding stage have been modified (e.g., deletion of the variable describing the environmental context, i.e., terrestrial or aquatic).

Finally, some deviations were specific to the literature from the search update:

- The search strategy over 2020–2023 was performed on the databases Web of Science Core Collection (WOS CC; on the Web of Science platform Clarivate) and Scopus (Elsevier), and on the search engine Google Scholar. CORE and BASE search engines were not searched because of limited resources of the map team.
- Abstract screening was performed using web-based software Abstrackr [22].

### Search strings

For consistency purpose, the exact same search strings used in Sordello et al. (2020) [13] were used for the search update [15]. The search string used to collect articles in the WOS CC database is presented below:

((TI = (noise OR sound$) OR TS = (“masking auditory” OR “man-made noise” OR “anthropogenic noise” OR “man-made sound$” OR “music festival$” OR ((pollution OR transportation OR road$ OR highway$ OR motorway$ OR railway$ OR traffic OR urban OR city OR cities OR construction OR ship$ OR boat$ OR port$ OR aircraft$ OR airplane$ OR airport$ OR industr* OR machinery OR “gas extraction” OR mining OR drilling OR pile-driving OR “communication network$” OR “wind farm$” OR agric* OR farming OR military OR gun$ OR visitor$) AND noise))) AND TS = (ecolog* OR biodiversity OR ecosystem$ OR “natural habitat$” OR species OR vertebrate$ OR mammal$ OR reptile$ OR amphibian$ OR bird$ OR fish* OR invertebrate$ OR arthropod$ OR insect$ OR arachnid$ OR crustacean$ OR centipede$))

Searches in Scopus were performed using the same string, but adapted to Scopus syntax. For searches on Google Scholar, the used search strings differ because of its specific search syntax. Thus, four search strings were built to preserve the global target of the original search string used on Scopus and WOS CC. Search strings used for Scopus and Google scholar are presented in Additional file 2. The comprehensiveness of the strings have already been tested, and details are available in Sordello et al. (2020) [13] and in the published protocol [17].

### Language

For consistency we followed the same decisions as in Sordello et al. (2020) [13]: (1) Searches were performed using exclusively English terms which enable literature written in any other language to be retrieved – based on the specificities of online publication database search engines–; (2) Only studies published in English or French were retained. The search of articles in other languages could not be made as we based it on the linguistic competences of the map team. Articles written in other languages than English or French were removed at full-text screening stage (*n* = 26). Therefore, we reckon it may constitute a potential bias in our systematic map.

### Search sources

The searches performed in Sordello et al. (2020) [13] cover a period ranging from 1932 to 2018 included and consider both peer-reviewed and grey literature. Searches were performed on two bibliographic databases: WOS CC and Scopus; and three web-based search engines: Google Scholar, BASE and CORE. They were also completed with a manual search on specialist websites and with a call for literature. After screening, they retained a total of 1794 relevant articles.

We updated the original literature base by performing a complementary search over 2020–2023 included. The search update was carried out on three literature sources: the two databases WOS CC and Scopus; and the search engine Google Scholar. These three bibliographic sources cover 92% of the relevant articles included in Sordello et al. (2020) [13], and we therefore considered them sufficient for updating purposes:

- The database Web of Science Core Collection (on the Web of Science platform Clarivate), using the access rights of the MNHN (French National Museum of Natural History). The search was run on 16 October 2023, and returned 3 291 citations.
- The database Scopus (Elsevier), using the access rights of the CNRS (the French National Centre for Scientific Research). The search was run on 16 October 2023, and returned 4 144 citations.
- The search engine Google Scholar. The search was run on 18 October 2023 through the software Publish or Perish v6 [23]. For each of the four search strings, the first 1 000 hits were exported, when available. Exports from the four search strings were merged, returning a total of 3 083 citations.

Citations from other minor sources were also included in the search update and treated jointly in the next steps:

- References from a complementary search performed by Sordello et al. (2020) [13] covering the years 2019 and 2020 (up to the 6 May 2020), that were not included in the first map (authors had screened but not coded them), to cover the year 2019 that is not included in the search update. 141 citations were included.
- References from an ongoing synthesis by Olivier Pichard, a member of the project’s expert committee. 191 citations were included.

Altogether, these sources cover a period from 1932 to October 2023.

### Screening process of the search update

The screening of eligible studies was carried out following predefined criteria, discussed with the map team and expert committee, and defined with regard to the PECO framework.

Firstly, citations were manually cleaned of duplicates (intra-duplicates and duplicates between sources) based on titles, authors and DOI matches using Microsoft Office Excel 2016 (duplicate conditional formatting and visual identification line by line). When available, citations that were kept were the ones from Scopus database because the metadata were more exhaustive compared to those from the WOS CC database and Google Scholar search results.

The screening process consisted of three stages: titles, abstracts and full-texts screening. We introduced alongside the traditional inclusion and exclusion categories a third classification category, labeled as “unclear”. This “unclear” category encompassed articles that fall on the periphery of the review scope or are too vague to warrant exclusion confidently. An article classified as “unclear” at title or abstract screening moved on to the next screening stage. An article classified as “unclear” at full-text screening was considered as definitively rejected (because the available information in the full-text did not allow a decision to be taken). The web-based software Abstrackr [22] was used to facilitate the abstract screening stage. Abstrackr is a collaborative platform that enables several reviewers to work in a shared space, and facilitates screening by highlighting discriminative keywords for inclusion and exclusion. The number of excluded articles at each stages is recorded in a ROSES flow diagram [24].

The screening process involved at least two reviewers at each stage. The involvement of at least two people in the article review process significantly enhances the reliability of the eligibility screening [25]. Titles were screened by two reviewers (RS and LT), abstracts by two reviewers (SV and LT), and full-texts by three reviewers (GD, RS and LT). We ensured that reviewers did not have to screen articles they authored themselves. At each stage, a Randolph Kappa test was conducted to evaluate consistency among reviewers before the screening starts. This test was applied to 5–10% of the corpus to be screened at each stage. To support consensus among reviewers, we considered as acceptable Kappa values above 0.7. Whatever the Kappa score, disagreements between reviewers were addressed and eligibility criteria were clarified when necessary. Subsequently, all articles underwent single screening.

### Citation chasing from relevant reviews

Additional references were incorporated from relevant reviews and meta-analyses included after the full-text screening stage of the search update. A review or meta-analysis was regarded as relevant if it met the following requirements:

- The whole paper met the scope of the PECO framework.
- Some parts of the paper met the scope of the PECO framework and the reviewed taxonomic groups and/or outcomes were underrepresented.

All citations referenced in the relevant reviews were extracted using the online application citationchaser, by one reviewer (BP) [26]. Citationchaser allows to automatically look for all records referenced in one or more articles and return a list of all referenced records. The list of chased citations was manually cleaned of duplicates within and duplicates previously searched (Sordello et al. (2020) [13] and the search update). Titles, authors and DOI matches were looked for using Microsoft Office Excel 2016 (duplicate conditional formatting and visual identification line after line). Chased citations followed the same screening process as previously described (see section: Screening process of the search update).

In order to identify candidates for a second round of citation chasing, reviews and meta-analyses issued from the first citation chasing went through a light screening process:

- Occurrences of the term “noise” were looked for with the Control Find command.
- Corresponding citations were deduplicate and screened as previously described.

Reviews and meta-analyses deemed relevant for the second round of citation chasing were submitted to the same steps as those in the first citation chasing process.

In the next steps, all citations collected from relevant reviews (first and second citation chasing) are treated alongside the citations from the search update.

### Refining process from Sordello et al. (2020) [13]

We refined articles included by Sordello et al. (2020) [13] to fit the scope of this updated systematic map: all articles related to fish, abstract noise and aquatic environments were removed. To this end, the map database underwent two refining processes: a filtering process and a new screening process.

Firstly, we filtered citations using Microsoft Office Excel 2016 on the basis of the metadata coded by Sordello et al. (2020) [13]. In this way we excluded citations where: (1) taxonomic group is prokaryotes, plants or fishes; or (2) exposure is abstract noise; or (3) environment is totally aquatic. We also excluded citations with PECO elements marked as “NA” or “Unknown” (meaning that the publication does not give the seeked information after full-text screening).

Secondly, we conducted an additional screening process directly on full-texts applying the eligibility criteria detailed below. Two reviewers performed screening (BP, LT), and a Randolph Kappa test using the method described above was performed between reviewers.

### Eligibility criteria

Articles were screened with regard to the PECO elements of the map question. Eligibility criteria are further detailed below.

- **Eligible population**: We included all wild animal terrestrial species (e.g., terrestrial mammals, reptiles, birds, arthropods, arachnids, etc.), semi-aquatic species (such as amphibians and pinnipeds), intertidal species (e.g., crabs, mussels), invertebrates (e.g., mollusks) and wild animals in captivity (e.g., zoo). We excluded humans, domestic/non-wild species (e.g., cats, dogs, laboratory rats, farm animals, etc.), strictly aquatic mammals (e.g., cetaceans), fishes, prokaryotes and unicellular organisms.
- **Eligible exposure**: We included ANN from all sources (e.g., transportation, urban, recreational), at any frequency (e.g., infrasound, ultrasound), of all temporality (e.g., continuous, intermittent, repeated), propagating in terrestrial environments (airborne noise), of all contexts and origins (spontaneous or recorded, in-situ or in laboratory, etc.). Noise in aquatic environment (underwater noise) was excluded. Any sounds produced by other animals (e.g., frog chorus) or natural events (e.g., rain, thunder) were excluded. “Abstract noises” such as experimental pings, tones or pulses were also disregarded, because animals are not exposed to them in practice. Sirens and alarms used as deterrents are intentionally disturbing to wildlife – rather than producing unintended or unknown impacts–, and were therefore not considered either.
- **Eligible comparator**: We considered all kinds of comparators: temporal comparator (conditions before and after exposure to ANN), spatial comparator (locations exposed and not exposed to ANN), gradients of exposure to disturbance.
- **Eligible outcome**: We considered all types of outcomes, including but not restricted to biology/physiology (e.g., heart rate), use of space (e.g., species distribution, occupancy), intra- and interspecific communication (e.g., song frequencies), species reproduction, community composition (e.g., species richness, abundance).
- **Eligible study design**: We included all experimental, observational and correlational studies. No particular restrictions were applied to study design (e.g., Control-Impact, Before-After Control-Impact).
- **Language**: Only articles published in English and in French were included.

At full-text screening stage, we included several additional criteria on information that is rarely available in titles and abstracts, which is why these criteria were not applied before.

- **Document type**: We included journal articles, theses, reports, books, chapter books and conference proceedings, formats which are likely to contain primary studies. Other formats were excluded (e.g., patents, press articles).
- **Document content**: We included only articles providing new primary knowledge (i.e., studies). Data papers were considered only if they contain data that is not published elsewhere. Modelling articles that only rely on simulated data were excluded. Reviews, meta-analyses, notes, letters, opinion papers, meeting abstracts, etc. were excluded. However, reviews and meta-analyses that meet our PECO criteria underwent a citation chasing process.
- **Exposure assessment**: We included articles where noise metrics were available (e.g., dB, Hz) whether assessed by authors, included from previous sources (e.g., database, monitoring, noise map) or easily inferable with a reliable proxy (e.g., road traffic, distance to windfarm). We also included articles where the exposure is controlled, i.e., studies in which researchers themselves induced noise exposure (e.g., playback of recorded noise). Research were the authors had extensive knowledge of the timing of the noisy event (e.g., fireworks) was considered as well. On the other hand, we excluded articles where noise difference between comparators was not quantified by noise measurements or by using a reliable proxy. Study designs that were not specifically built to isolate the impact of noise were also excluded. In such cases, it remains uncertain whether noise characteristics truly differed between the compared contexts, and therefore if the study is actually about noise. Studies only relying on acoustic indices (e.g., Acoustic Diversity Index, Acoustic Complexity Index, Normalized Difference Soundscape Index) to characterize noise were also excluded. That is because eco-acoustic indicators aim principally at characterizing soundscape structure, or biodiversity through biophony, not the level of ANN [27]. To this end, the amplitude of the signal is usually normalized before any further processing, resulting in the loss of any indication, even relative, of sound intensity or frequency. Therefore, these articles deviate from our original question.

### Coding strategy

**Fig. 1:**
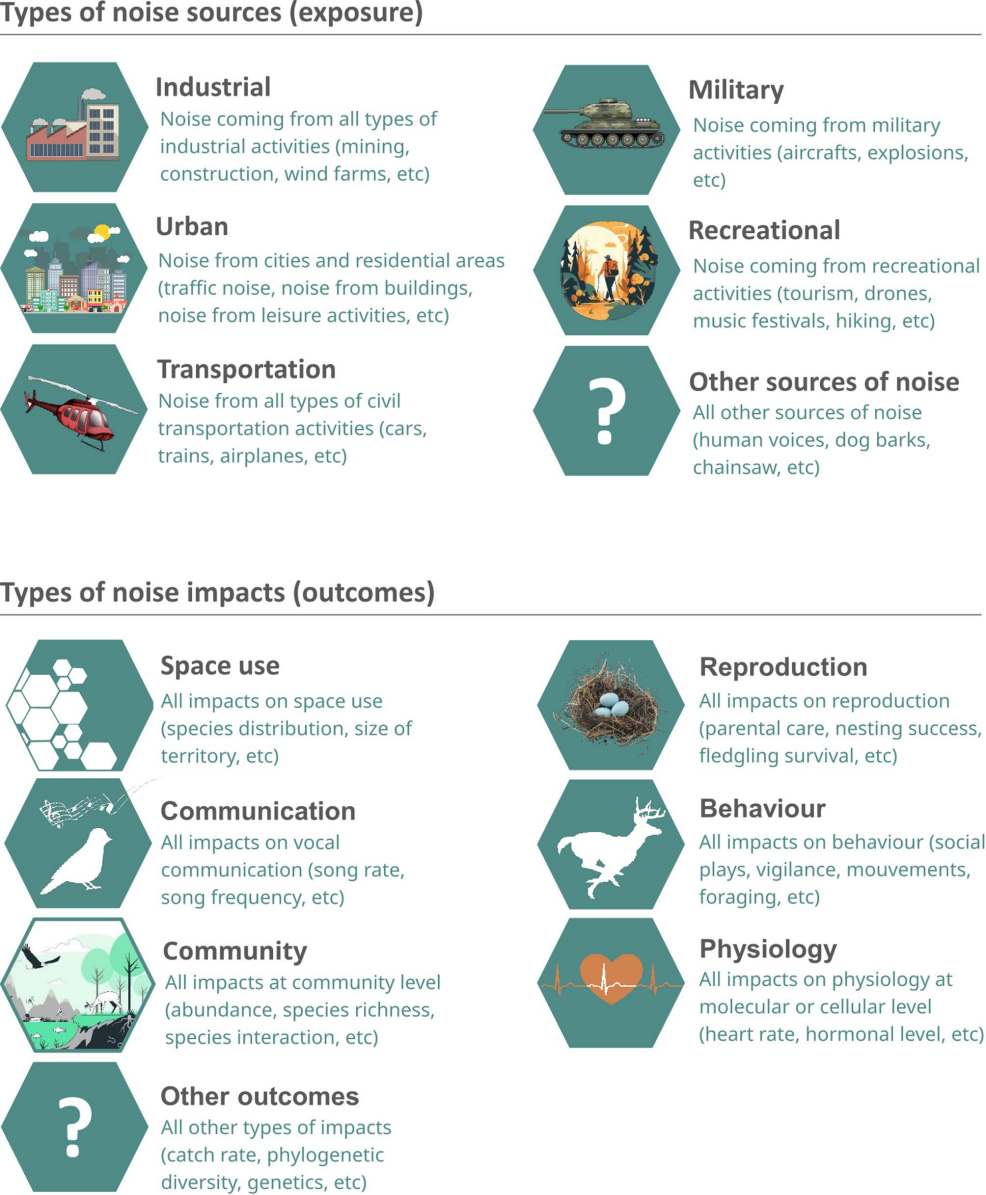
Categories used to code sources of noise (exposure) and impacts of noise on wildlife (outcomes)

Articles from the original map by Sordello et al. (2020) [13] have already undergone coding. For consistency purpose, we applied the same coding strategy to articles from the search update. Coding was carried out on full-texts, using a pre-designed datasheet, featuring coded options to ensure standardized and consistent data extraction. Coding fields rely on keywords and expanded comments to describe various aspects. The codebook includes key variables related to:

- Bibliographic information (source, DOI, language, authors, container, title, year of publication, document type and document content);
- Articles characteristics (taxonomic group, noise source, outcome, research team location, study location), categories for types of exposure and outcomes are further detailed in Fig. 1;
- Study context (in-situ / ex-situ) and design (experimental / observational).

Four coders (LT, BP, PA, CF) performed the coding of the updated literature. A random sample of 15 articles (∼ 7% of the update database) underwent double-checking to ensure consistency among the coders. Disagreements were discussed and solved, and when required, the codebook was revised accordingly. The coders also discussed together any difficult cases during the coding process with the possibility of involving experts from the review team when deemed relevant. During metadata extraction, the missing or unclear information were coded as such. We only coded primary studies. Reviews and meta-analyses were not coded.

### Study validity assessment

Mapping the quality of available literature was not a goal of this systematic map. As a result, in accordance with CEE guidelines for systematic maps, we did not perform any validity assessment of the included articles. Further systematic reviews on knowledge clusters will enable to provide such critical appraisal.

### Data-mapping method

We proposed a synthetic overview of the overall literature base, encompassing both the literature from Sordello et al. 2020 [13] and the updated literature. Figures were produced to show the proportion of articles type and content, the locations of studies, the chronological distribution of publications and the evolution of methodological designs over time. We also described studies according to the key elements of their question (taxonomic groups, noise sources and outcomes). Knowledge gaps and clusters were identified based on the number of articles and relevance to stakeholders in order to advise primary research directions and guide future reviews. We chose to define knowledge clusters based on taxonomic groups and outcomes that form biologically coherent clusters, rather than on exposure which reflects a more human-centered perspective.

The literature was mapped at article level (not study level). When articles were associated with multiple labels (in terms of content or key elements), they were duplicated to ensure that each label corresponds to a single entry. For example, an article that covers two taxonomic groups of birds and mammals (which translates in the database as ‘Birds| Mammals’) was split into two lines so that each tag could be considered individually (one line labelled ‘Birds’ and the other ‘Mammals’). In this way, each label is equally considered, whenever the article is associated with unique or multiple labels.

All figures were produced using the free software R (R Core Team 2024) [28] and the libraries *tidyverse* [29], *dplyr* [30],*ggplot2* [31], *ggforce* [32], *ggraph* [33], *igraph* [34], *maps* [35], *countrycode* [36], *stringr* [37], *sf* [38], *viridis* [39], *fmsb* [40], *corrplot* [41] and *circlize* [42].

## Review findings

### Literature search and screening process

**Fig. 2:**
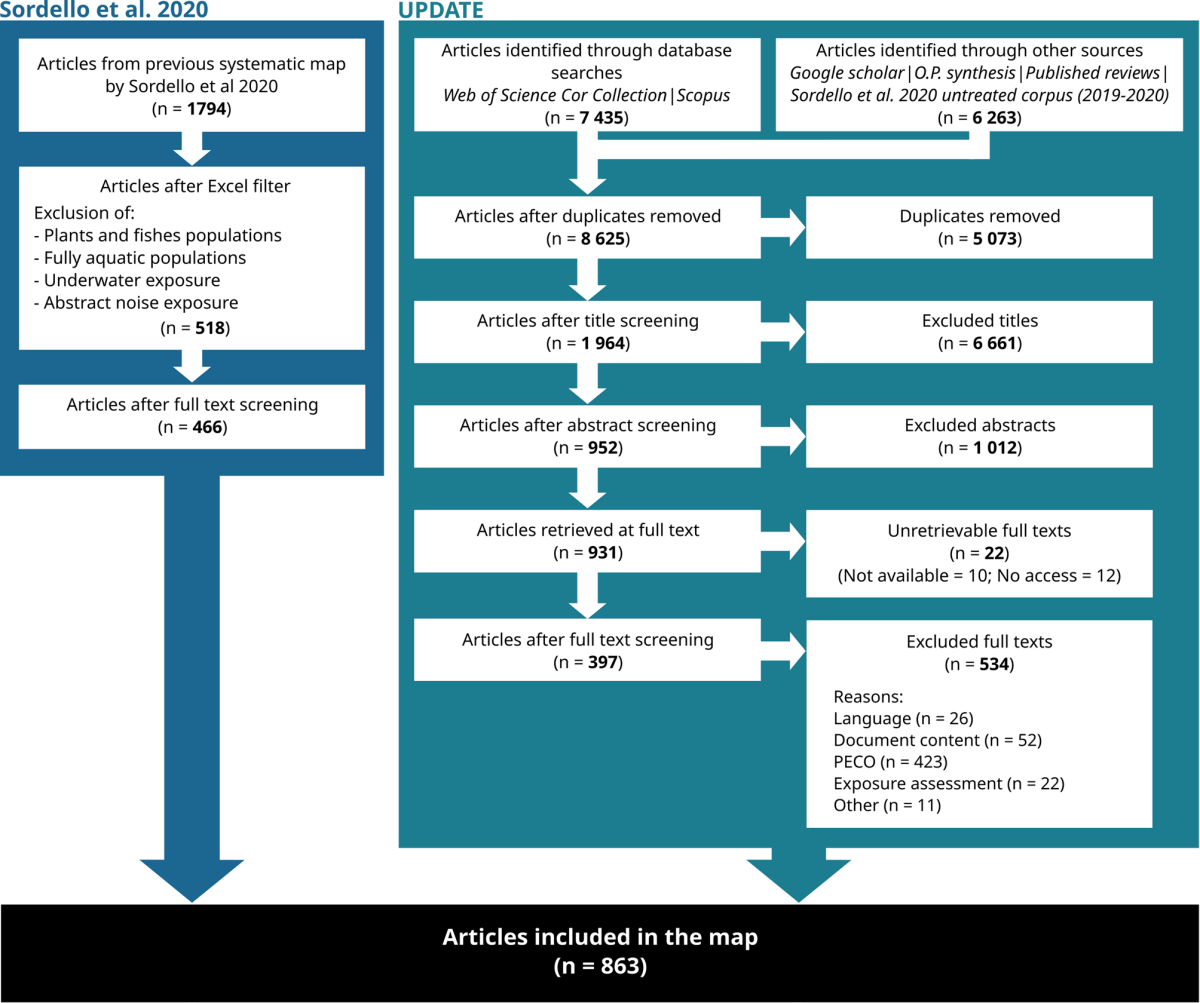
ROSES flow diagram of the systematic map, including the literature from Sordello et al. (2020) [13] and the literature from the update

An overview of the screening process is provided by the ROSES flow diagram (Fig. 2) which shows the volumes at the different stages.

The search update collected a total of 13 698 citations. Among them, 5 073 were removed because they were duplicates. Over screening stages, 6 661 were excluded at title screening, 1 012 at abstract screening, 22 full-texts were not retrieved and 534 were excluded at full-text screening. In the end, 397 relevant articles from the search update were included in the systematic map. Detailed screening at each stage (title, abstract and full text stages) of the updated literature is available in Additional file 3, along with the 51 reviews used for citation chasing. The list of excluded articles at full-text screening is provided in Additional file 4, along with explanations for their exclusion. Coded data of the primary studies from the update are available in Additional file 5.

The record from Sordello et al. 2020 [13] provided a total of 1 794 articles. After applying the Excel filter based on the coded data to remove articles whose taxonomic groups and/or noise characteristics did not fall within our revised scope, we obtained 518 articles. At the end of the new full-text screening, 466 relevant articles from Sordello et al. 2020 [13] were included in the systematic map. The results of this full-text screening are provided in Additional file 6 and the coded data (primary studies) are available in Additional file 7.

In total, 863 relevant articles are included in the systematic map (the complete systematic map database is available in Additional file 8).

### Articles sources

Articles included in the systematic map come from two main sources:

- Literature from Sordello et al. 2020 [13], 466 articles, 54%.
- Literature from the update, 397 articles, 46%.

The origin of the articles from the search update is distributed as follows:

- Bibliographic databases Scopus (226 articles, 57%) and WOS CC (28 articles, 7%), 254 articles, 64%.
- Search engine Google Scholar, 33 articles, 8%.
- Sordello et al. 2020 [13] untreated corpus (2019–2020), 39 articles, 10%.
- Synthesis authored by Olivier Pichard, 15 articles, 4%.
- References from relevant reviews (i.e. citation chasing), 56 articles, 14%.

Bibliographic databases are the most relevant sources of bibliography in the search update. Sordello et al. 2020 [13] made a similar observation.

### Article types and contents

**Fig. 3:**
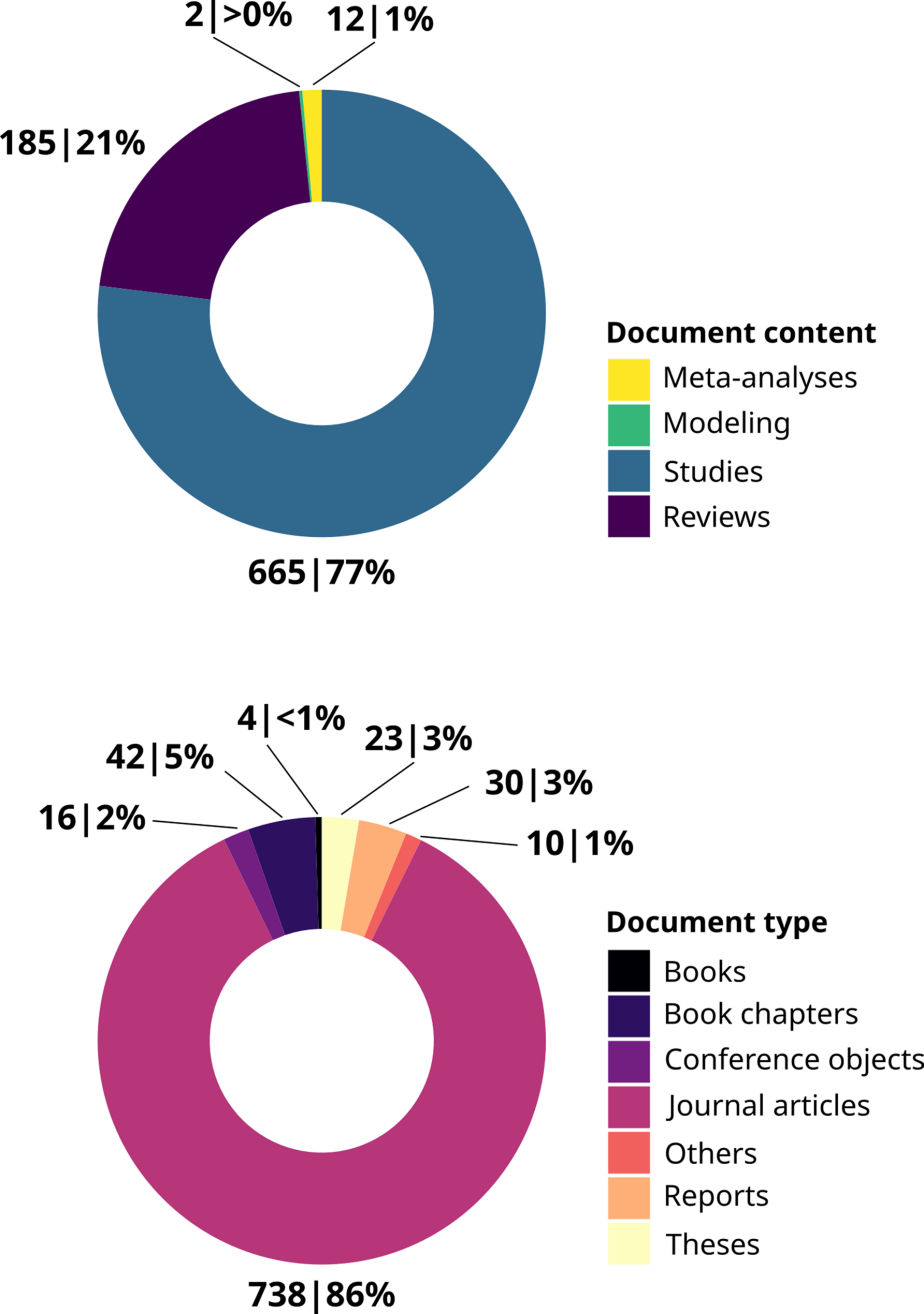
Contents (top) and types (bottom) of articles included in the systematic map (number of articles|percentage of the total)

Distribution of article *contents* is presented in Fig. 3 (top). The majority of articles are primary studies (665 articles, 77%), but reviews are the second main important content (181 articles, 21%). On the other hand, the literature base contains very few meta-analyses and modelling papers, each accounting for 1% or less of all articles.

The distribution of article *types* is showed in Fig. 3 (bottom). Most studies are published as journal articles (738 articles, 86%). The topic has been the subject of several books and book Chaps. (46 articles, 5%) and PhD theses (23 articles, 3%), underlying the conceptual interest of the scientific community for the topic of ANN.

### Study location

**Fig. 4:**
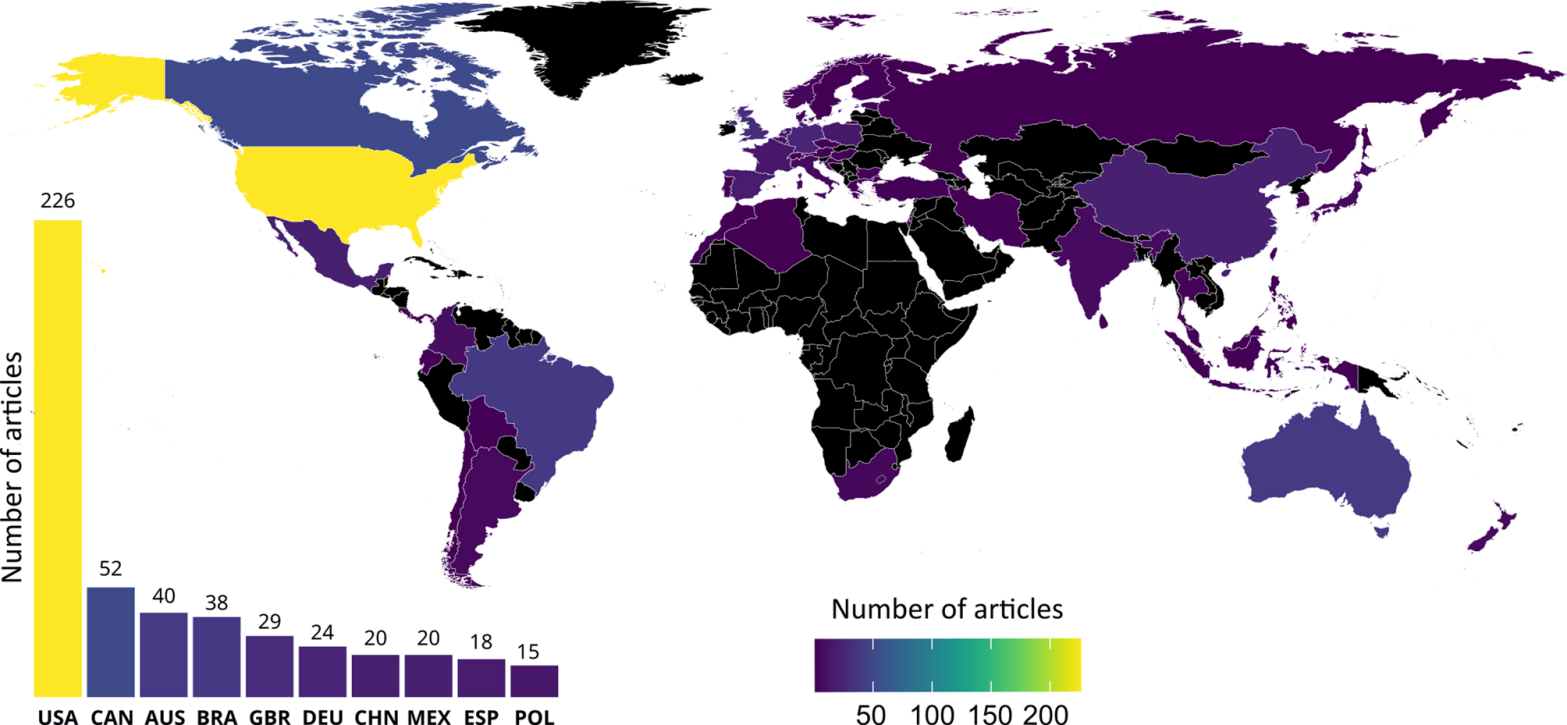
Geographical distribution of the articles included in the systematic map

A total of 59 countries are covered by the systematic map. Figure 4 displays the locations of primary research studies. The United States (USA) produced 226 articles, which represent 34% of our record. Canada (CAN) was the second more prolific country (52 studies, 8%), closely followed by Australia (AUS; 40 studies, 6%) and Brazil (BRA; 38 studies, 6%).

### Chronological distribution

**Fig. 5:**
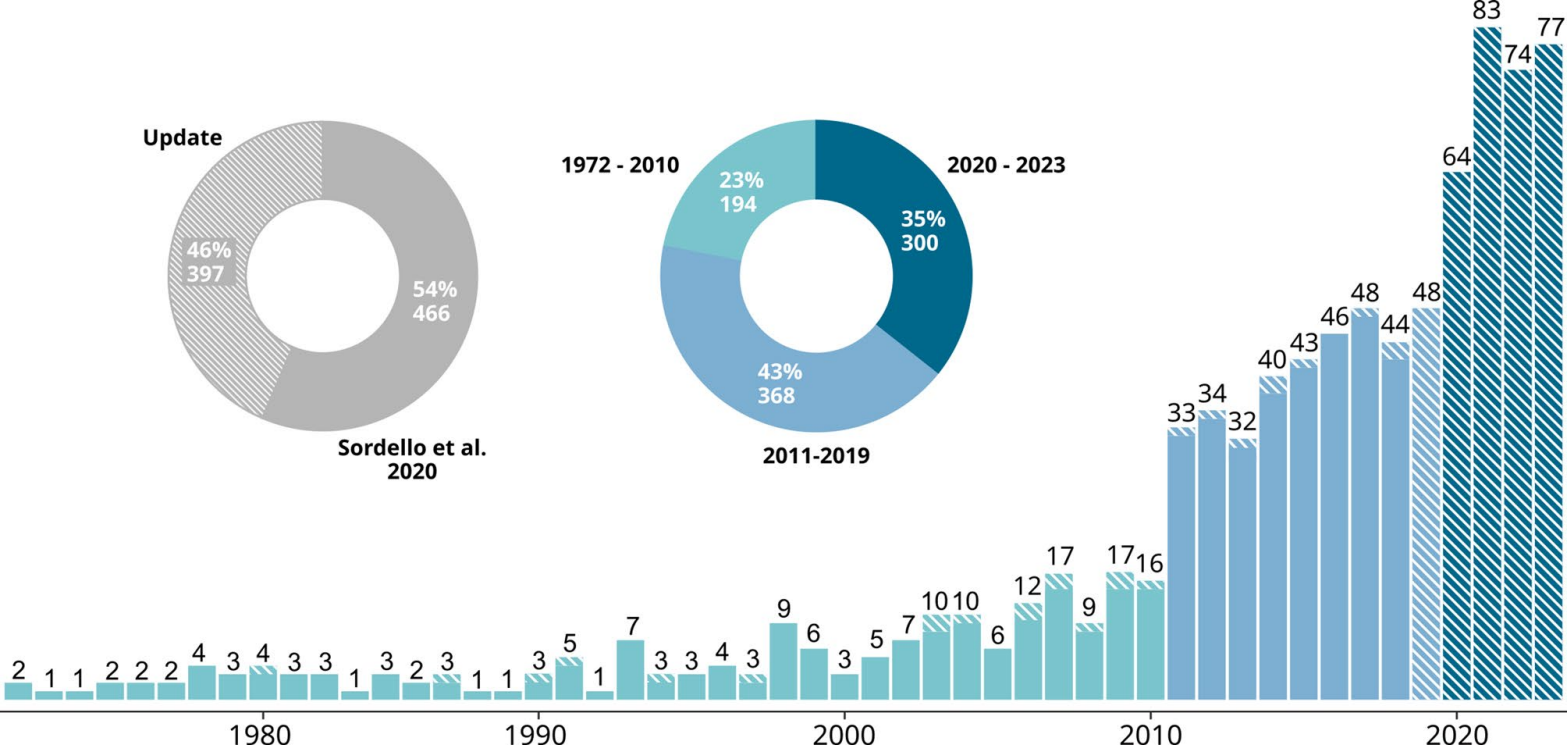
Chronological distribution of articles until October 2023. The respective contributions of the literature from the search update and from Sordello et al. (2020) [13] are indicated by hatching. Three periods of low, medium and high interest for the topic are visually identified and displayed in shades of blue

The systematic map database contains articles from 1972 to 2023. Figure 5 illustrates the respective contribution of the results from the search update and from Sordello et al. 2020 [13] to the systematic map, and shows how the production of articles has varied over the years. The publication rate has increased sharply over time. However, this trend is observed across all research topics and primarily reflects the rise of systematic online archiving of articles (making them increasingly accessible), publication inflation (driven by the pressure to publish, researchers are producing more articles each year), as well as technological developments that facilitate fieldwork (lower energy consumption, lower storage costs, lower hardware costs). Nevertheless, the graph shows two unusual notable shifts – one in 2011 and another in 2020 – reflecting the scientific community’s growing interest in ANN. Three distinct periods can thus be visually identified, with the annual number of articles nearly doubling between each phase:

- A period of low interest for the topic before 2011, when publication rate was of one to 15 articles per year;
- A period of medium interest for the topic between 2011 and 2019, when publication rate was of ∼ 30 to 45 articles per year;
- A period of high interest for the topic after 2019, when publication rate was of ∼ 60 to 75 articles per year.

### Evolution of document contents and study designs over time

**Fig. 6:**
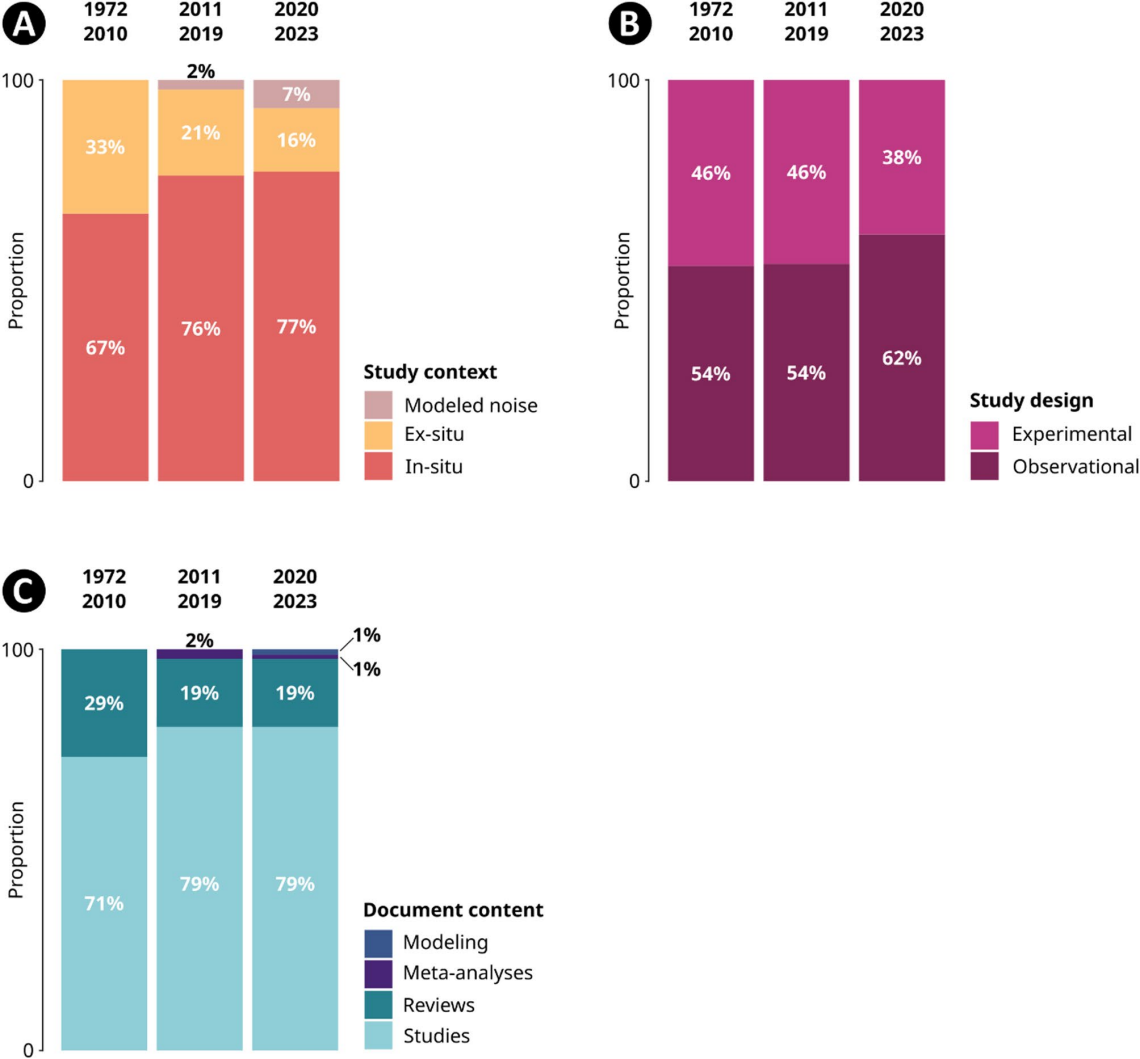
Proportional variation in study context (**A**) and study design (**B**), and in document content (**C**) between period of low, medium and high interest

In proportion, more reviews were published over the low interest period (1972–2010; 29% of all articles; Fig. 6c) compared to following periods (both 19% of all articles; Fig. 6c). The medium interest period (2011–2019) witnessed the publication of the first meta-analyses on the topic, and the high interest period (2020–2023) the first modelling articles. As interest in the subject grew, new forms of knowledge syntheses emerged and modelling approaches offered novel conceptual insights into the processes underlying ANN.

Among primary studies, the share of in-situ studies has risen over time (67% in the period of low interest, 76% in the period of medium interest, 77% in the period of high interest; Fig. 6a), while the share of ex-situ studies decreased (33% in the period of low interest, 21% in the period of medium interest, 16% in the period of high interest; Fig. 6a). In the medium interest period, the first articles based on modelled noise were published, reaching 7% of all studies in the period of high interest. Modelled noise refers to approaches such as noise propagation modelling and mapping, which rely on point-based measurements to simulate noise levels over broader geographical areas.

The proportion of studies using observational designs increased during the high interest period (62% of studies, compared to 54% before; Fig. 6b) and a decrease in experimental studies (38% of studies, compared to 46% before; Fig. 6b). Observational designs refer to non-interventional studies in which conclusions are drawn from correlations or covariation between variables (e.g., studies comparing biodiversity in an airport with a natural forest according to level of noise), whereas experimental designs involve deliberate manipulation of noise by the research team (e.g., broadcasting road noise to create a “phantom road” applying a before/after design). Observational designs are considered less powerful than experimental designs because they reveal patterns rather than processes. However, they are currently regaining popularity in ecology because they allow large-scale studies to be conducted and they have recently been enhanced by technological advances [43]. The increasing availability of large databases of occurrences and life history traits from scientific and participatory projects in recent years may explain this trend.

### Distribution of the literature by taxonomic groups, noise sources and outcomes

**Fig. 7:**
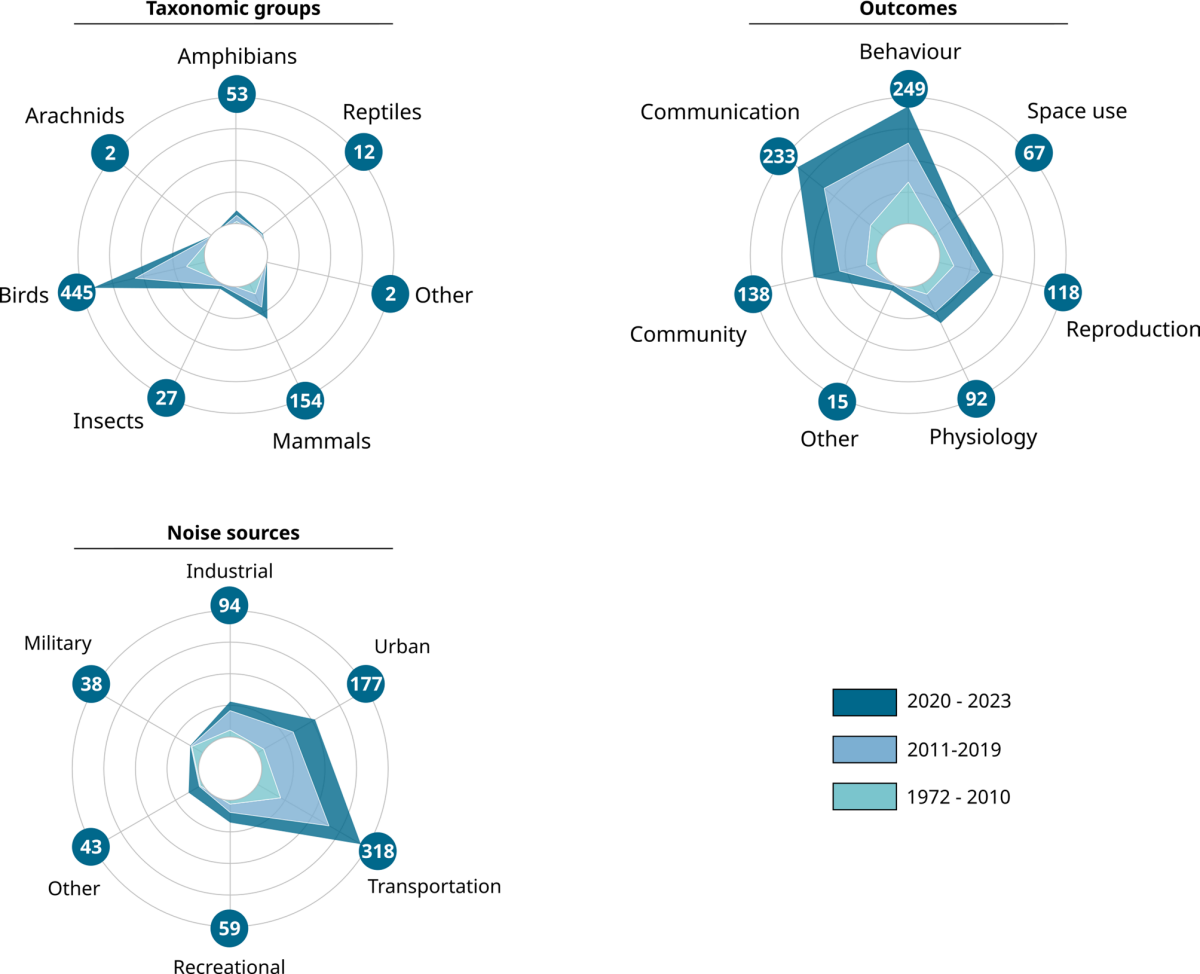
Number of articles for each taxonomic group, outcome and noise source

The far more studied taxonomic group is birds (445 articles, representing 64% of studies; Fig. 7a), distantly followed by mammals (154 articles, representing 22% of studies; Fig. 7a). This trend is shared by the three periods of low, medium and high interest, and has become more pronounced over time.

Behaviour is the more commonly examined outcome response (249 articles, representing 27% of studies; Fig. 7b), closely followed by communication (233 articles, representing 25% of studies; Fig. 7b) and then by outcomes at community level (139 articles, representing 15% of studies; Fig. 7b). During the period of low interest (1972–2010), the scientific community focused almost exclusively on behavioural impacts, only to diversify its interest towards communication in later periods.

The most widely studied source of ANN is transportation (318 articles, representing 43% of studies; Fig. 7c), followed by urban noise (177 articles, representing 24% of studies; Fig. 7c). Other noise sources remain currently under-studied, each accounting for less than 12% of studies. During the period of low interest (1972–2010), there was a particular focus on military noise, making it the second most studied noise source at that time. However, this type of noise source received less attention in later periods, likely overshadowed by transportation noise, which is considered the primary source of ANN. As the primary research activities in this area are associated with military activities, the related publications may not be available to the public.

**Fig. 8:**
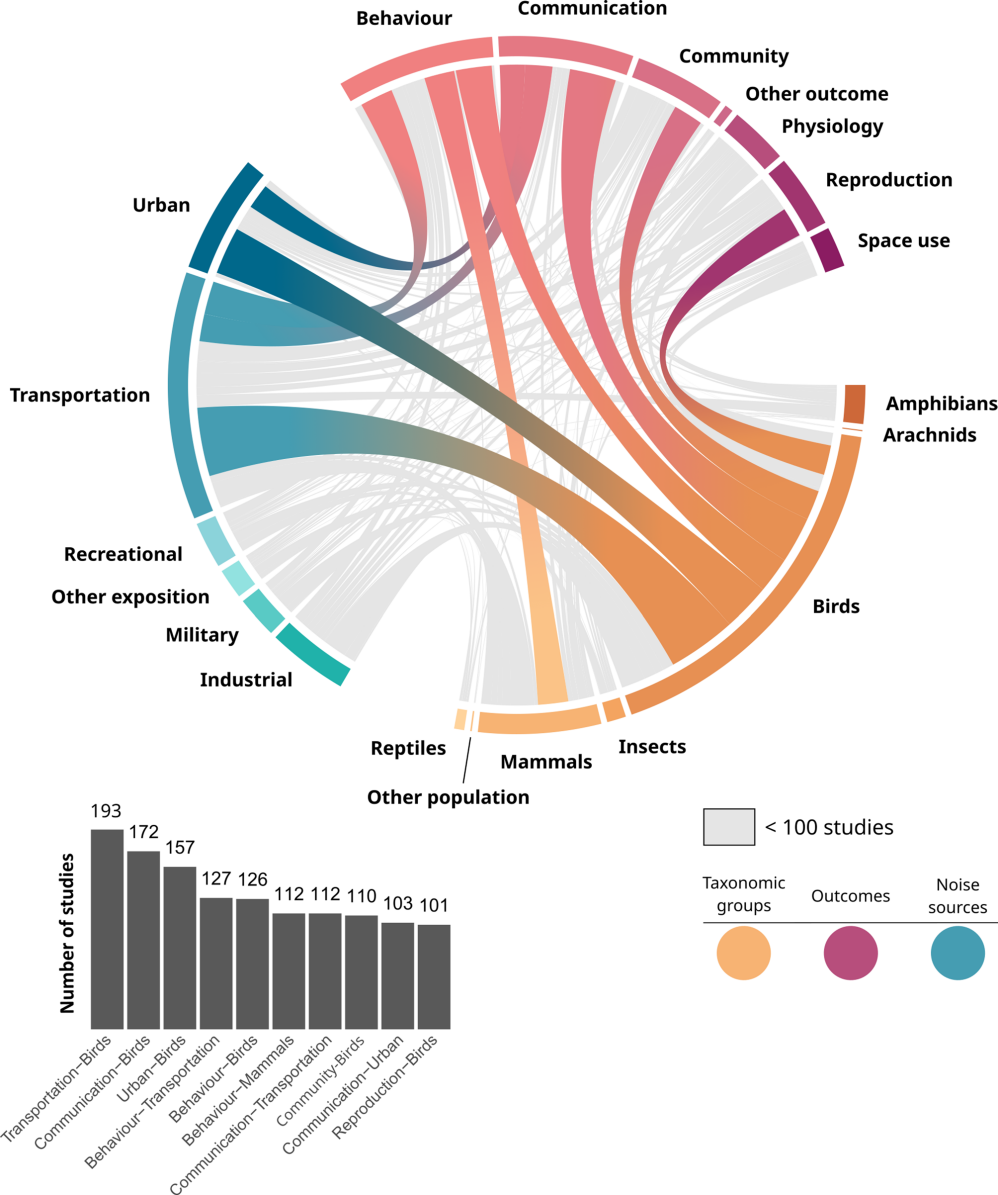
Chord diagram showing the number of studies addressing each pair of key elements (taxonomic groups, outcomes and noise sources)

Not only are noise sources, taxonomic groups and outcomes unevenly studied individually, but their combined examination within studies also lacks consistency (Fig. 8). Among all combinations of taxonomic groups, noise sources and outcomes, ten pairs are well-studied and are investigated together in at least 100 studies. This threshold was selected based on a noticeable gap in pair occurrences (between 101 and 76 occurrences; see Additional file 9), which appeared to us as a meaningful divide between commonly and less commonly studied pairs of key elements. The most commonly studied pair is Transportation-Birds (282 studies), followed by Urban-Birds (195 studies) and Birds-Communication (191 studies). Only one pair concerns another taxonomic group than birds with more than 100 occurrences: Mammals-Behaviour. Crossed tables detailing the number of studies associated with each pair of key elements are available in Additional file 9.

### Knowledge clusters

**Fig. 9:**
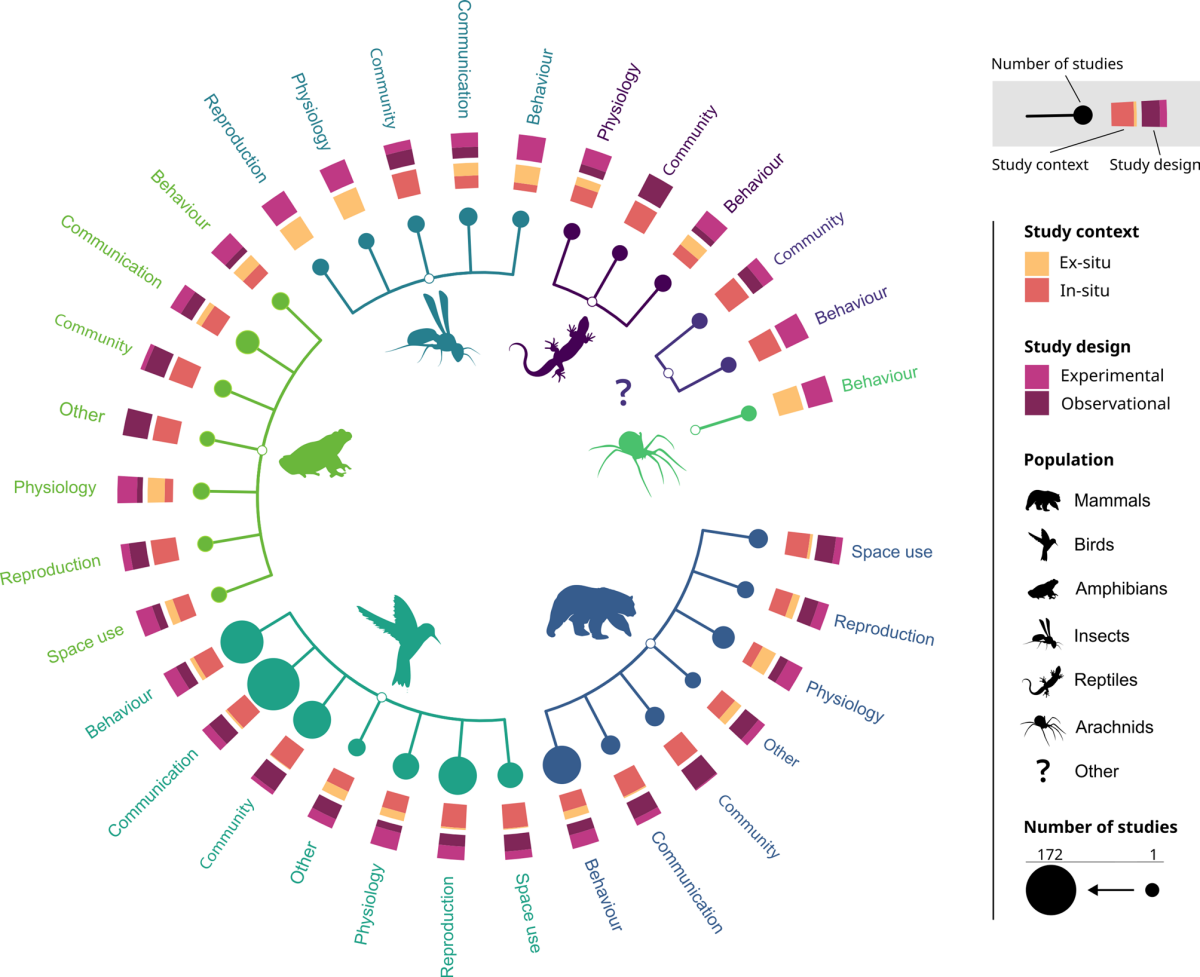
Knowledge clusters defined by taxonomic groups and outcomes. The number of studies per potential cluster is represented, along with detailed representation of study contexts and designs within each cluster

Each intersection between key elements (Fig. 8) warrants attention. Here, we considered a knowledge cluster suitable for review if it comprises a substantial number of studies and addresses a question that has not yet been synthesized. Five clusters of knowledge, based on pairs of taxonomic group and outcome, appeared to be well represented in the literature: Birds-Behaviour, Birds- Communication, Birds-Community, Birds-Reproduction and Mammals-Behaviour (Fig. 9). Two of these clusters have already been extensively synthetized: Birds-Communication (e.g. [44–48]), and Birds-Behaviour (e.g [47, 49, 50]). To guide primary research, we highlighted three clusters of knowledge that may deserve particular attention:

1. How does airborne anthropogenic noise impacts mammals’ behaviour?
  A first knowledge cluster of 112 studies addresses the first most studied outcome (behaviour) and the second most studied taxonomic group (mammals). This cluster offers an opportunity to explore the impact of ANN on another taxonomic group than birds that are by far the most studied and reviewed taxonomic group. As behaviour is one of the first response of organisms to disturbance, these 112 studies could be the focus of a systematic review to investigate the primary mechanisms underlying the impacts of ANN. However, this cluster includes a wide range of sub-outcomes (time allocation, vigilance, flee, hide, etc.) which may prove difficult to be meta-analyzed.
2. How does airborne anthropogenic noise impacts birds’ reproductive success?
  This second cluster of 101 studies addresses the most studied taxonomic group (birds). This cluster has already been reviewed (e.g [51, 52]). but it would benefit from a more comprehensive synthesis and, if possible, meta-analyses. It raises important conservation issues since reproductive success is directly related to fitness and then to species persistence. Thus, this cluster could be the focus of a systematic review; however, it also includes several sub-outcomes (e.g., number of eggs, number of fledglings, offspring viability).
3. How does airborne anthropogenic noise impacts wildlife community level?
  This final cluster comprises 138 studies. By including all populations, it offers a broad perspective on the impacts of ANN on communities, encompassing species interactions, diversity, and abundance. While it is conceptually valuable to consider all taxonomic groups when examining community-level responses, it is important to note that the majority of studies focus on birds. We therefore leave it to future reviewers to decide whether to restrict the scope of this cluster to avian taxa. Outcomes contain metrics such as abundance and species richness that are easy to be meta-analyzed. Thus, this cluster could be the focus of a relevant systematic review exploring the deep and long-term impact of ANN on communities’ dynamic.

### Knowledge gaps

#### Study location

The African continent is clearly understudied, with only five studies from South Africa, two from Morocco and one from Algeria (Fig. 4). Central Asia would also benefit from further investigations.

#### Taxonomic groups

Studies show a strong focus on birds and mammals (86% of studies), while other vertebrates such as reptiles (2% of studies) or invertebrates such as insects (4% of studies) and arachnids (< 1% of studies) are clearly understudied (Fig. 7). Only one article was found on soil fauna such as earthworm [53]. We found no studies on the impacts of ANN on intertidal species (e.g., oysters, mussels), and very few on pinnipeds (4 studies), even though these marine semi-aquatic organisms are exposed to air for a significant part of their daily cycle.

#### Outcomes

Outcomes are studied unevenly between taxonomic groups: all types of ANN impacts are the subject of at least one study in birds and mammals, whereas only few are addressed in reptiles and insects (Additional file 9). Conversely, almost all combinations of outcomes and noise sources are addressed. Consequences of ANN on reproduction have been studied a lot in birds (86% of studies on reproduction), but is poorly addressed in other taxonomic groups, which is an unneglectable knowledge gap considering the major role of reproduction in species persistence. Space use is also quite understudied and has not been assessed at all in certain organisms such as reptiles, insects and arachnids. Studying space use would be relevant, as ANN triggers significant avoidance and flight behaviours, which could lead to changes in occupancy at a larger scale.

#### Noise sources

Exposure is strongly focused on noise coming from transportation and urban areas, while noise from military, recreational and industrial sources is less represented, in particular in the last years (since 2020). Urban and transportation noise typically originates from human activity centers (e.g., roads, cities), whereas recreational, military, and industrial activities are often located deeper within natural environments. As a result, they can impact more remote locations such as national parks or natural reserves, likely to harbor species that do not adapt well to the artificialization and anthropization of their environments. There is therefore a major knowledge gap in assessing the impact of ANN in wild and remote areas.

### Limitations of the map

Our methodology is based on the systematic map published by Sordello et al. (2020) [13] in 2020, therefore the limitations they discussed in their publication also apply to our work. This includes limitations related to the search strategy as we only considered two academic databases, which is considered as the minimum to according to CEE guidelines [14]. Additionally, for the search update we used only three of the bibliographic sources originally used by Sordello et al. (2020) [13] (i.e. WOS CC, Scopus and Google Scholar). However, as these three bibliographic sources cover 92% of the articles considered relevant and included in Sordello et al. (2020) [13], we therefore assumed they were sufficient for updating purposes. Also, because of resource limitation, we were not able to perform double screening and double coding on all articles, so there may have been some misclassification errors. To circumvent this issue, as it is allowed by CEE guidelines, a set of articles (5–10% of the corpus) was used to test inter-reviewers’ agreement prior to each stage, so we are confident that this risk is minimal. Finally, we only accepted articles written in English or French, while over full-text screening of the search update we came across several articles written in Chinese, Spanish or Arabic. However, these articles only represent 0.03% of articles that were screened at full-text (26 over 931 articles) and they are easily accessible in the additional files to be screened by another team if relevant.

A specific limitation relates to coding. The literature from Sordello et al. (2020) [13] and the literature from the search update were not coded by the same team, so there may be differences in the way the coding method was applied. To minimize this bias, we used the exact same coding method and CodeBook as the ones used by Sordello et al. (2020) [13].

Although, as previously explained, we defined knowledge clusters based on taxonomic groups and outcomes to form biologically coherent categories, rather than using noise sources, which reflect a more human-centered perspective. We acknowledge that this choice may be a limitation, as stakeholders often find it more practical to develop prevention measures based on noise sources rather than taxonomic groups. We therefore encourage reviews adopting that perspective to prioritize clusters defined by taxonomic groups and noise sources.

Finally, a last limitation concerns the identification of knowledge gaps. Some important gaps related to noise properties — such as frequency or temporal patterns — may have gone unnoticed, as these variables were not coded in this map. These finer details may be important to address in future reviews.

## Conclusion

This systematic map catalogues 863 documents published from 1972 to 2023 about the impacts of ANN on wildlife, including 665 articles of primary research. As a result, it provides an updated and comprehensive overview on the available literature worldwide. This literature has already been the subject of several syntheses (196 reviews and meta-analyses). However, subtopics remain treated unevenly. In particular, three clusters of knowledge have been identified as suitable candidates for future systematic reviews: (1) How does ANN impacts mammals’ behaviour? (2) How does ANN impacts birds’ reproductive success? (3) How does ANN impacts birds at community level?

### Implication for policy/management

ANN is a major concern in society, but particularly in terms of public health. Indeed, noise has various harmful impacts on human health [54, 55] and international organizations have been tackling this issue, sometimes for several decades [56–58]. Conversely, concerns about ecological impacts of noise are still evolving very slowly. However, this systematic map highlights – following on from Sordello et al. (2020) [13] – that a huge amount of the existing literature could be transferred to mitigation or protection measures (after thorough assessment of the literature). ANN would need to be considered in a very large number of sectors; e.g., land and transport planning, regulating certain activities, changing industrial processes. When reviews are intended to inform stakeholders, we encourage authors to consider focusing on clusters defined by noise sources rather than by taxonomic groups and outcomes, depending on stakeholder needs and priorities. Noise sources reflect a more human-centered perspective, which is often more practical for developing targeted prevention and mitigation measures. We therefore recommend that reviews aimed at supporting stakeholder decision-making prioritize clusters organized by noise sources.

Today, more and more scientists are calling for the inclusion of ANN in biodiversity conservation policies. Sensory pollutants such as noise, light and odors are gaining a lot of impetus as a global issue to tackle [59]. Practitioners and designers in ecology are invited to integrate sound, light and volatile compounds, taking into account both natural and artificial stimuli [60]. For example, the concept of ‘quiet areas’ has been introduced by the European Union in a directive on environmental noise [61, 62]. It is now recommended to preserve and restore cores but also corridors taking account of these sensory disturbances [63]. Such an investigation needs knowledge that may be found in the literature gathered by this systematic map (e.g., sensitivity thresholds [64]). Nevertheless, light pollution is still much more considered at operational and political level than ANN. As a result, it would be relevant to pay more attention to the latter, which involves other challenges for ecosystems and life on Earth. In contrast to light, noise is not easily stopped by obstacles in the line of sight. Regarding noise, until now, most attention has been focused on marine ecosystems – rightly given the impacts of noise on cetaceans in particular – but this systematic map stresses that a large quantity of studies also exists on terrestrial ecosystems that would deserve as much attention and whose level of literature should be evaluated.

### Implication for research

Compared to the previous systematic map by Sordello et al. (2020) [13], our results suggest that existing trends have intensified: knowledge clusters have grown denser, while knowledge gaps became wider in comparison. As a result, despite the increasing attention paid to ANN research, many taxonomic groups are still understudied (especially reptiles and arachnids), many potential sources of ANN are underrepresented (e.g., recreational and military noise) and outcomes are unevenly investigated between taxonomic groups (e.g., impacts on reproduction are poorly studied in reptiles). We believe that the knowledge gaps identified in this systematic map can be used to guide future research and to address the evident imbalance in the existing literature. Some of these gaps represent a serious lack for biodiversity conservation whether in terms of biomass (e.g., arthropods represent a major part of ecosystems) or activities (e.g., regarding the trendy development of ecotourism).

Regarding secondary research, we encourage authors interested in conducting a review and/or meta-analysis on one of the identified knowledge clusters to consider incorporating noise properties such as frequency and temporal patterns. Although these variables were not coded in the present map, they represent important dimensions of noise exposure that may influence wildlife responses differently. Addressing these finer-scale characteristics in future syntheses could provide more nuanced insights into the effects of ANN.

Moving one step further, there are several aspects of the question that would deserve attention from primary research. First, there is a lack of studies in the literature that take a functional perspective, meaning that studies preferentially focus taxonomic groups rather than on organisms sharing traits related to ANN tolerance (e.g [65]). Functional approaches are complementary to taxonomic approaches and could provide information on the features that can make a species more or less vulnerable to noise disturbance. Secondly, very few studies have investigated the long-term impacts of ANN on morphological and/or functional features (e.g. body size [66, 67]). The evolution of acoustic traits and of the morphofunctional features that mediate their production or reception are often linked (e.g [68, 69]). Investigating these questions could provide insights into the evolutionary pressures generated by ANN and their consequences for the evolution of species. Lastly, another direction for research would be to address the vibrational component of noise transmitted through the ground or surfaces, as opposed to the acoustic component of noise. We found only one study that considered anthropogenic vibrations [53], although our search string may not contain the adequate keywords to gather articles related to vibrational noise.

In recent years, an increasing number of studies based on participatory databases and noise maps have been published (e.g [70–72]). These approaches provide more statistical power to assess large-scale patterns between ANN and wildlife. Yet, too few modelling studies have been carried out to understand the evolutionary mechanisms underlying these patterns. As demonstrated by this systematic map update, ANN is an issue of growing interest within the scientific community, and studies on this topic are expected to increase in the coming years. To maintain an up-to-date overview of the state of the art, it would be beneficial to regularly update this systematic map.

## Electronic supplementary material

Below is the link to the electronic supplementary material.

Additional file 1: ROSES for Systematic Map Reports

Additional file 2: Search strings for each search support and citations counts per string

Additional file 3: Screening stages of the literature update

Additional file 4: List of excluded full texts with reason for rejection of the literature update

Additional file 5: Coding table of the literature update

Additional file 6: Re-screening of the literature from Sordello et al. 2020

Additional file 7: Coding table of the literature from Sordello et al. 2020

Additional file 8: Systematic map database

Additional file 9: Crossed tables detailing the number of studies associated with each pair of key elements (taxonomic groups, outcomes, noise sources)

## Data Availability

All data generated or analysed during this study are included in this published article [and its supplementary information files].

## References

[1] Bradbury JW, Vehrencamp SL. Principles of animal communication. 2nd ed. Sinauer Associates; 2011.

[2] Romer H, Bailey W. Insect hearing in the field. Camp Biochem Physiol. 1990;97:443–7.

[3] Dominoni D, Smit JAH, Visser ME, Halfwerk W. Multisensory pollution: artificial light at night and anthropogenic noise have interactive effects on activity patterns of great Tits (Parus major). Environ Pollut. 2020;256:113314. 31761596 10.1016/j.envpol.2019.113314

[4] Mason JT, McClure CJW, Barber JR. Anthropogenic noise impairs Owl hunting behavior. Biol Conserv. 2016;199:29–32.

[5] Tilgar V, Hein K, Viigipuu R. Anthropogenic noise alters the perception of a predator in a local community of great Tits. Anim Behav. 2022;189:91–9.

[6] Ditmer MA, Vincent JB, Werden LK, Tanner JC, Laske TG, Iaizzo PA, et al. Bears show a physiological but limited behavioral response to unmanned aerial vehicles. Curr Biol. 2015;25:2278–83. 26279232 10.1016/j.cub.2015.07.024

[7] Matyjasiak P, Chacińska P, Książka P. Anthropogenic noise interacts with the predation risk assessment in a free-ranging bird. Møller A, editor. Current Zoology. 2023;zoad019. 10.1093/cz/zoad019 PMC11255998 39035757

[8] Schou CPE, Levengood AL, Potvin DA. Limited effects of traffic noise on behavioural responses to conspecific mating calls in the Eastern sedge frog Litoria fallax. Acta Ethol. 2021;24:217–26. 34366558 10.1007/s10211-021-00378-7 PMC8335461

[9] Merrall ES, Evans KL. Anthropogenic noise reduces avian feeding efficiency and increases vigilance along an urban–rural gradient regardless of species’ tolerances to urbanisation. J Avian Biol. 2020;51:jav02341.

[10] Ortiz-Jiménez L, Iglesias-Merchan C, Barja I. Behavioral responses of the European Mink in the face of different threats: conspecific competitors, predators, and anthropic disturbances. Sci Rep. 2021;11:8266. 33859346 10.1038/s41598-021-87905-5 PMC8050081

[11] Longcore T, Rich C, Mineau P, MacDonald B, Bert DG, Sullivan LM, et al. Avian mortality at communication towers in the united States and canada: which species, how many, and where? Biol Conserv. 2013;158:410–9.

[12] Tyack PL, Zimmer WMX, Moretti D, Southall BL, Claridge DE, Durban JW et al. S Thrush editor 2011 Beaked whales respond to simulated and actual navy sonar. PLoS ONE 6 e17009. 10.1371/journal.pone.0017009 PMC3056662 21423729

[13] Sordello R, Ratel O, Flamerie De Lachapelle F, Leger C, Dambry A, Vanpeene S. Evidence of the impact of noise pollution on biodiversity: a systematic map. Environ Evid. 2020;9:20.

[14] Collaboration for Environmental Evidence. 2022. Guidelines and Standards for Evidence synthesis in Environmental Management. Version 5.1 (AS Pullin, GK Frampton, B Livoreil & G Petrokofsky, Eds) www.environmentalevidence.org/information-for-authors. [02/11/2023].

[15] Bayliss HR, Haddaway NR, Eales J, Frampton GK, James KL. Updating and amending systematic reviews and systematic maps in environmental management. Environ Evid. 2016;5:20.

[16] One Health Joint Plan of Action, 2022–2026. Rome: FAO; UNEP, WHO.; World Organisation for Animal Health (WOAH) (founded as OIE); 2022 Oct. Available from: http://www.fao.org/documents/card/en/c/cc2289en

[17] Sordello R, Flamerie De Lachapelle F, Livoreil B, Vanpeene S. Evidence of the environmental impact of noise pollution on biodiversity: a systematic map protocol. Environ Evid. 2019;8:8.

[18] Pullin AS. Updating reviews: commitments and opportunities. Environ Evid. 2014;3:18.

[19] Guan S, Brookens T. An overview of research efforts to understand the effects of underwater sound on cetaceans. Water Biology Secur. 2023;2:100141.

[20] Pieniazek RH, Beach RK, Dycha GM, Mickle MF, Higgs DM. Navigating noisy waters: A review of field studies examining anthropogenic noise effects on wild fish. J Acoust Soc Am. 2023;154:2828–42. 37930177 10.1121/10.0022254

[21] Haddaway NR, Macura B, Whaley P, Pullin A. ROSES for systematic review protocols. Version 1.0. 2017.

[22] Wallace BC, Small K, Brodley CE, Lau J, Trikalinos TA. Deploying an interactive machine learning system in an evidence-based practice center: abstrackr. Proc of the ACM International Health Informatics Symposium (IHI). 2012;819–24.

[23] Harzing AW. Publish or Perish. 2007. Available from: https://harzing.com/resources/publish-or-perish

[24] Haddaway NR. ROSES_flowchart(): An R package and ShinyApp. 2020.

[25] Frampton GK, Livoreil B, Petrokofsky G. Eligibility screening in evidence synthesis of environmental management topics. Environ Evid. 2017;6:27.

[26] Haddaway NR, Grainger MJ, Gray CT, Citationchaser. An R package and Shiny app for forward and backward citations chasing in academic searching (0.0.3). Zenodo. 2021; Available from: 10.5281/zenodo.4543513

[27] Bradfer-Lawrence T, Gardner N, Bunnefeld L, Bunnefeld N, Willis SG, Dent DH. Guidelines for the use of acoustic indices in environmental research. Methods Ecol Evol. 2019;10:1796–807.

[28] R Core Team. R: A Language and Environment for Statistical Computing. Vienna, Austria: R Foundation for Statistical Computing. 2024. Available from: https://www.R-project.org/

[29] Wickham H, Averick M, Bryan J, Chang W, McGowan LD, François R, et al. Welcome to the tidyverse. J Open Source Softw. 2019;4:1686.

[30] Wickham H, François R, Henry L, Müller K, Vaughan D. dplyr: A Grammar of Data Manipulation. 2023. Available from: https://CRAN.R-project.org/package=dplyr

[31] Wickham H. ggplot2: Elegant Graphics for Data Analysis. Springer-Verlag New York; 2016. Available from: https://ggplot2.tidyverse.org

[32] Pedersen TL. ggforce: Accelerating ggplot2. 2024. Available from: https://CRAN.R-project.org/package=ggforce

[33] Pedersen TL. ggraph: An Implementation of Grammar of Graphics for Graphs and Networks. 2024. Available from: https://CRAN.R-project.org/package=ggraph

[34] Csárdi G, Nepusz T, Traag V, Horvát S, Zanini F, Noom D et al. igraph: Network Analysis and Visualization in R. 2024. Available from: https://CRAN.R-project.org/package=igraph 10.1371/journal.pone.0355108 PMC13436765 42550872

[35] Becker OS code by, RA. Minka ARWR version by RBE by TP, Deckmyn A. maps: Draw Geographical Maps. 2023. Available from: https://CRAN.R-project.org/package=maps

[36] Arel-Bundock V, Enevoldsen N, Yetman CJ. Countrycode: an R package to convert country names and country codes. J Open Source Softw. 2018;3:848.

[37] Wickham Hstringr. Simple, Consistent Wrappers for Common String Operations. 2023. Available from: https://CRAN.R-project.org/package=stringr

[38] Pebesma E. Simple features for R: standardized support for Spatial vector data. R J. 2018;10:439–46.

[39] Garnier S, Ross, Noam, Rudis R et al. viridis(Lite) - Colorblind-Friendly Color Maps for R. 2024. Available from: https://sjmgarnier.github.io/viridis/

[40] Nakazawa M. fmsb: Functions for Medical Statistics Book with some Demographic Data. 2024. Available from: https://CRAN.R-project.org/package=fmsb

[41] Wei T, Simko V. R package corrplot: Visualization of a Correlation Matrix. 2024. Available from: https://github.com/taiyun/corrplot

[42] Gu Z, Gu L, Eils R, Schlesner M, Brors B. Circlize implements and enhances circular visualization in R. Bioinformatics. 2014;30:2811–2. 24930139 10.1093/bioinformatics/btu393

[43] Sagarin R, Pauchard A. Observational approaches in ecology open new ground in a changing world. Front Ecol Environ. 2010;8:379–86.

[44] Marín-Gómez OH, MacGregor‐Fors I. A global synthesis of the impacts of urbanization on bird dawn choruses. Ibis. 2021;163:1133–54.

[45] Oden AI, Brandle JR, Burbach ME, Brown MB, Gerber JE, Quinn JE. Soundscapes and anthromes: A review of proximate effects of traffic noise on avian vocalization and communication. 2020;5–5:203–8.

[46] Derryberry EP, Luther D. What is Known—and not Known—About acoustic communication in an urban soundscape. Integr Comp Biol. 2021;61:1783–94. 34124755 10.1093/icb/icab131

[47] Singh G, Sharma, G, Kumar S, Chaudhary K. Anthropogenic noise affect the bird song frequency and behavioral response. 2023.

[48] Nemeth E, Pieretti N, Zollinger SA, Geberzahn N, Partecke J, Miranda AC, et al. Bird song and anthropogenic noise: vocal constraints May explain why birds Sing higher-frequency songs in cities. Proc R Soc B. 2013;280:20122798. 10.1098/rspb.2012.2798 PMC3574330 23303546

[49] Fossesca M, Henry KS, Chou TL, Gall MD. The silent assumption of the masking hypothesis: avian auditory processing and implications for behavioral responses to anthropogenic noise. Front Ecol Evol. 2023;11:1233911.

[50] Blumstein DT. Attention, habituation, and antipredator behaviour: implications for urban birds. In: Gil D, Brumm H, editors. Avian urban ecology. Oxford University Press; 2014.

[51] Reijnen R, Foppen R, Veenbaas G. Disturbance by traffic of breeding birds: evaluation of the effect and considerations in planning and managing road corridors. Biodivers Conserv. 1997;6:567–81.

[52] Chen S, Liu Y, Patrick SC, Goodale E, Safran RJ, Pagani-Núñez E. A multidimensional framework to quantify the effects of urbanization on avian breeding fitness. Ecol Evol. 2023;13:e10259. 37404704 10.1002/ece3.10259 PMC10316489

[53] Velilla E, Collinson E, Bellato L, Berg MP, Halfwerk W. Vibrational noise from wind energy-turbines negatively impacts earthworm abundance. Oikos. 2021;130:844–9.

[54] Hahad O, Kuntic M, Al-Kindi S, Kuntic I, Gilan D, Petrowski K et al. Noise and mental health: evidence, mechanisms, and consequences. J Expo Sci Environ Epidemiol. 2024 [cited 2024 Feb 8]; Available from: https://www.nature.com/articles/s41370-024-00642-5 10.1038/s41370-024-00642-5 PMC11876073 38279032

[55] Mehrotra A, Shukla SP, Shukla AK, Manar MK, Singh SK, Mehrotra M. A Comprehensive Review of Auditory and Non-Auditory Effects of Noise on Human Health. Noise and Health. 2024 [cited 2024 Oct 25];26:59–69. Available from: https://journals.lww.com/10.4103/nah.nah_124_23 10.4103/nah.nah_124_23 PMC11530096 38904803

[56] World Health Organization. Guidelines for community noise. 1999 p. 141. Report No.: WHO Reference Number: a68672. Available from: https://www.who.int/publications-detail-redirect/a68672

[57] Programme des Nations Unies pour l’environnement. Bruit, flammes et décalages. Questions émergentes d’ordre environnemental. Nairobi. 2022 p. 59.

[58] Commission Européenne. La politique future de luttre contre le bruit. 1996 p. 48. Available from: https://eur-lex.europa.eu/LexUriServ/LexUriServ.do?uri=COM:1996:0540:FIN:FR:PDF

[59] Halfwerk W, Slabbekoorn H. Pollution going multimodal: The complex impact of the human-altered sensory environment on animal perception and performance. Biology Letters. 2015;11. Available from: https://www.scopus.com/inward/record.uri?eid=2-s2.0-84929120516%26doi=10.1098/rsbl.2014.1051%26partnerID=40%26md5=28105728977a478c0d6930241de44054 10.1098/rsbl.2014.1051 PMC4424613 25904319

[60] Elmer LK, Madliger CL, Blumstein DT, Elvidge CK, Fernández-Juricic E, Horodysky AZ et al. Exploiting common senses: sensory ecology meets wildlife conservation and management. Franklin C, editor. Conservation Physiology. 2021 [cited 2022 Nov 3];9:coab002. Available from: https://academic.oup.com/conphys/article/doi/10.1093/conphys/coab002/6199346 10.1093/conphys/coab002 PMC8009554 33815799

[61] European Environment Agency. Quiet areas in Europe — The environment unaffected by noise pollution. 2014. Available from: https://www.eea.europa.eu/publications/quiet-areas-in-europe

[62] DIRECTIVE 2002/49/EC OF THE EUROPEAN PARLIAMENT AND OF THE COUNCIL of 25. June 2002 relating to the assessment and management of environmental noise. Official J Eur Communities. 2002.

[63] Sordello R. Écologie du paysage et écologie sensorielle: prendre en compte les pollutions lumineuses, sonores et olfactives dans les trames écologiques. De la connaissance à l’action [phdthesis]. Museum National d’Histoire Naturelle; 2024 [cited 2024 Oct 25]. Available from: https://hal.science/tel-04645145

[64] Moulherat S, Plotard C, De Roincé C, Cornuau J. Prendre en compte la pollution sonore dans une modélisation de dynamiques de populations d’espèces. SET. 2023 [cited 2023 Dec 9];43–7. Available from: https://revue-set.fr/article/view/7512

[65] Pereyra LC, Akmentins MS, Salica MJ, Quiroga MF, Moreno CE, Vaira M. Tolerant and avoiders in an urban landscape: Anuran species richness and functional groups responses in the yungas’ forest of NW Argentina. Urban Ecosyst. 2021;24:141–52.

[66] Kirk EC, Gosselin-Ildari AD. Cochlear labyrinth volume and hearing abilities in Primates. Anat Rec. 2009;292:765–76. 10.1002/ar.20907 19462443

[67] Gleich O, Dooling RJ, Manley GA. Audiogram, body mass, and Basilar papilla length: correlations in birds and predictions for extinct archosaurs. Naturwissenschaften. 2005;92:595–8. 16231131 10.1007/s00114-005-0050-5

[68] Corfield JR, Krilow JM, Vande Ligt MN, Iwaniuk AN. A quantitative morphological analysis of the inner ear of galliform birds. Hear Res. 2013;304:111–27. 23871766 10.1016/j.heares.2013.07.004

[69] Borgard HL, Baab K, Pasch B, Riede T. The shape of sound: a geometric morphometrics approach to laryngeal functional morphology. J Mammal Evol. 2020;27:577–90.

[70] Injaian AS, Francis CD, Ouyang JQ, Dominoni DM, Donald JW, Fuxjager MJ et al. Baseline and stress-induced corticosterone levels across birds and reptiles do not reflect urbanization levels. Cooke S, editor. Conservation Physiology. 2020;8:coz110. 10.1093/conphys/coz110 PMC6978728 31993201

[71] Klingbeil BT, La Sorte FA, Lepczyk CA, Fink D, Flather CH. Geographical associations with anthropogenic noise pollution for North American breeding birds. Sheard C, editor. Global Ecol Biogeogr. 2020;29:148–58.

[72] Senzaki M, Barber JR, Phillips JN, Carter NH, Cooper CB, Ditmer MA, et al. Sensory pollutants alter bird phenology and fitness across a continent. Nature. 2020;587:605–9. 33177710 10.1038/s41586-020-2903-7

