# Supplementary material for: What is the evidence for the impacts of airborne anthropogenic noise on wildlife? A systematic map update
Source: Environ Evid. 2025 Jul 26;14:14. doi: 10.1186/s13750-025-00368-3 (PMC12297495; doi:10.1186/s13750-025-00368-3)
Supplement: Supplementary file 2 — Additional file 2: Search strings for each search support and citations counts per string [file 13750_2025_368_MOESM2_ESM.docx]

**Additional file 2: Search strings**

**Databases:**

**Web of Science Core Collection**

The search string building process was conducted on the Web of Science Core Collection database. For additional details about this procedure, refer to Sordello et al. (2020) (Additional file 2). The resulting search string is as follows:

((TI = (noise OR sound$) OR TS= (“masking auditory” OR “man-made noise” OR “anthropogenic noise” OR “man-made sound$” OR “music festival$” OR ((pollution OR transportation OR road$ OR highway$ OR motorway$ OR railway$ OR traffic OR urban OR city OR cities OR construction OR ship$ OR boat$ OR port$ OR aircraft$ OR airplane$ OR airport$ OR industr* OR machinery OR “gas extraction” OR mining OR drilling OR pile-driving OR “communication network$” OR “wind farm$” OR agric* OR farming OR military OR gun$ OR visitor$) AND noise))) AND TS = (ecolog* OR biodiversity OR ecosystem$ OR “natural habitat$” OR species OR vertebrate$ OR mammal$ OR reptile$ OR amphibian$ OR bird$ OR fish* OR invertebrate$ OR arthropod$ OR insect$ OR arachnid$ OR crustacean$ OR centipede$))

For update purpose, the search was run over the period 2020 to 2023.

> Results and exports for the search update: 3 291 exported citations on 16/10/2023.

**Scopus**

For Scopus, we adapted the search string used for Web of Science Core Collection to take into account differences in search syntax. As previously, the search was restricted to years 2020 to 2023. It resulted in the following search string:

( TITLE ( noise OR sound ) OR TITLE-ABS-KEY ( "masking auditory" OR "man-made noise" OR "anthropogenic noise" OR "man-made sound" OR "music festival" OR ( ( pollution OR road OR highway OR motorway OR railway OR traffic OR urban OR city OR cities OR ship OR boat OR aircraft OR airport OR airplane OR industr* OR "gas extraction" OR mining OR drilling OR pile-driving OR "wind farm" OR visitor OR port OR agric* OR farming OR construction OR military OR transportation OR machinery OR gun OR "communication network" ) AND noise ) ) ) AND ( TITLE-ABS-KEY ( ecolog* OR biodiversity OR ecosystem OR "natural habitat" OR species OR vertebrate OR mammal OR reptile OR amphibian OR bird OR fish* OR invertebrate OR arthropod OR insect OR arachnid OR crustacean OR centipede ) )

> Results and exports for the search update: 4 144 exported citations on 16/10/2023.

**Search engines**

**Google Scholar**

To maintain consistency with the systematic map search protocol (Sordello et al. 2020), we used Publish or Perish v6 software (Harzing 2007).

- Search modalities:

Search was performed:

- on years 2020 to 2023,

- on all languages,

- excluding patents,

- including citations.

Results were sorted by citation frequency.

- Search strings:

New search strings had to be designed to adapt to Google Scholar's search syntax. Four search strings were built to maintain the overall purpose of the original search string used on Scopus and Web of Science Core Collection :

- Search string 1:

All of the words: animal

Any of the words: "music festival" "masking auditory” "man-made sound" "masking auditory" "man-made noise" "anthropogenic noise"

- Search string 2:

All of the words: noise

Any of the words: ecology ecological biodiversity ecosystem "natural habitat" species vertebrate mammal reptile amphibian bird fish invertebrate arthropod insect arachnid crustacean centipede

- Search string 3:

All of the words: “noise pollution”

Any of the words: transportation road highway motorway railway traffic urban city cities construction ship boat port aircraft airplane airport

- Search string 4:

All of the words: “noise pollution”

Any of the words: industrial machinery "gas extraction" mining drilling pile-driving "communication network" "wind farm" agriculture farming military gun visitor

> Results and exports per search strings for the search update:

Search string 1: all results exported (83 citations)

Search string 2: first 1 000 results exported

Search string 3: first 1 000 results exported

Search string 4: first 1 000 results exported

On 18/10/2023.

The four exports were merged (3 083 citations).
