# Supplementary material for: What is the evidence for the impacts of airborne anthropogenic noise on wildlife? A systematic map update
Source: Environ Evid. 2025 Jul 26;14:14. doi: 10.1186/s13750-025-00368-3 (PMC12297495; doi:10.1186/s13750-025-00368-3)
Supplement: Supplementary file 9 — Additional file 9: Crossed tables detailing the number of studies associated with each pair of key elements (taxonomic groups, outcomes, noise sources) [file 13750_2025_368_MOESM9_ESM.docx]

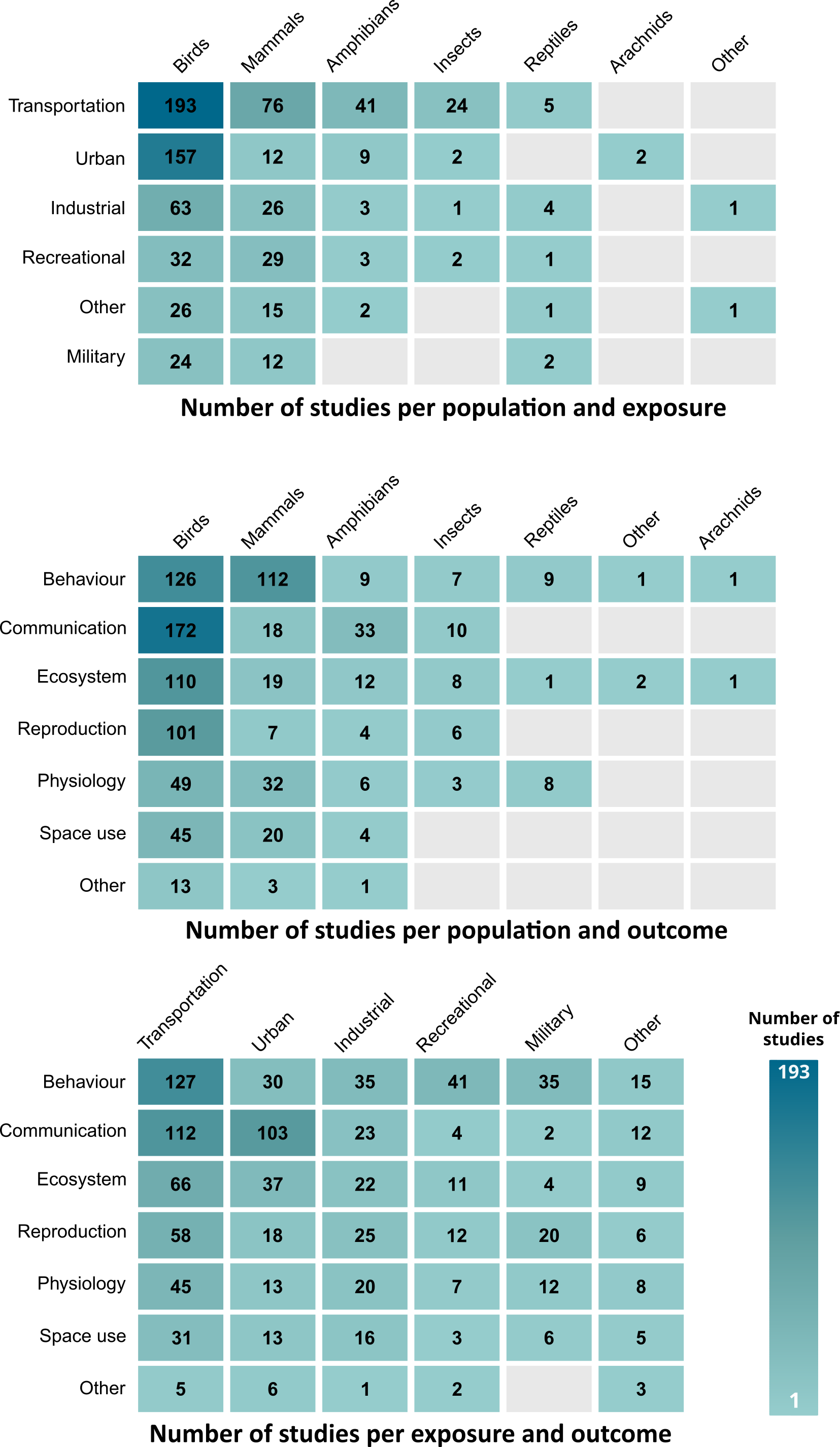


**Number of studies per taxonomic groups and noise sources**


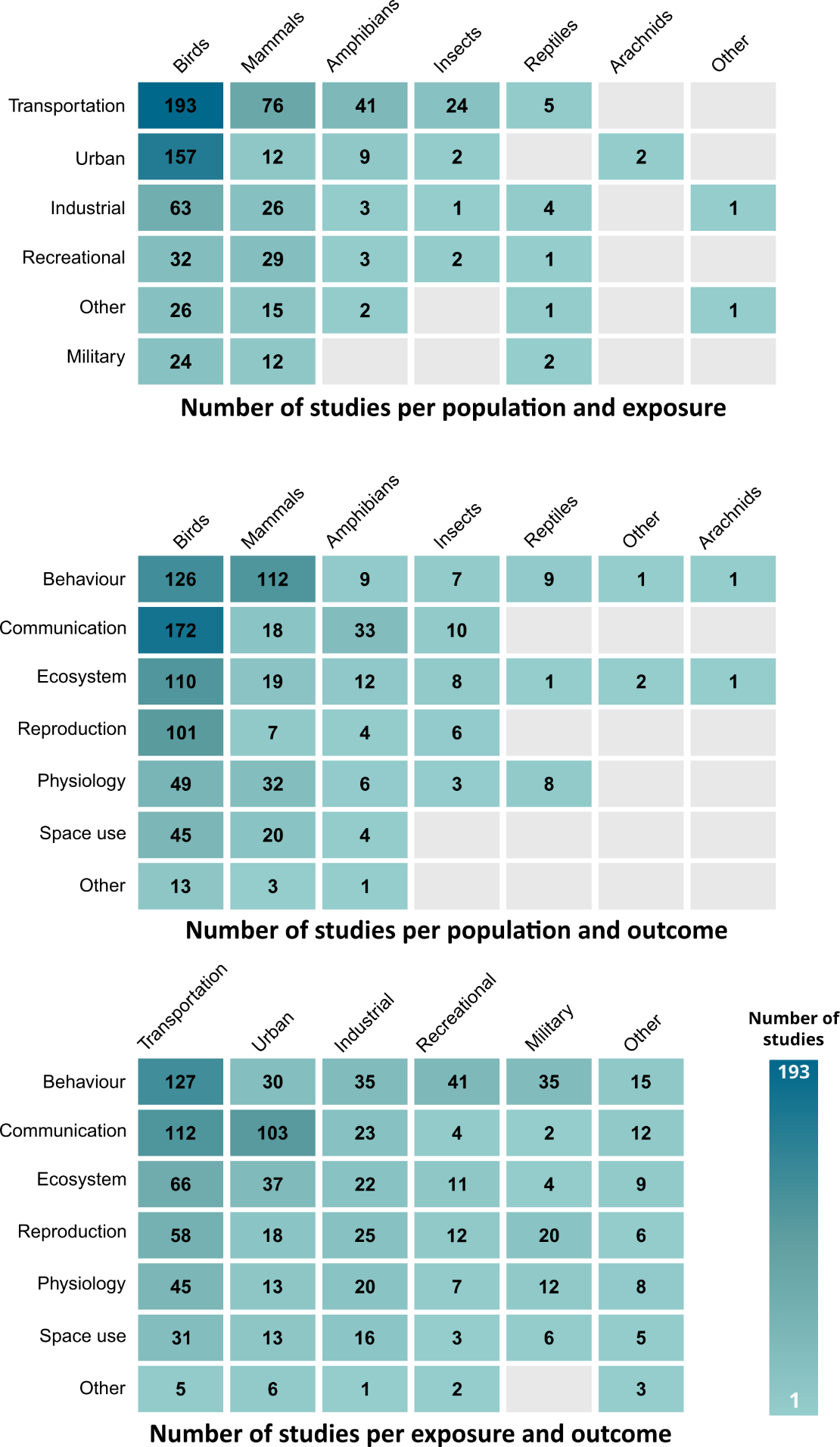


**Number of studies per taxonomic groups and outcomes**


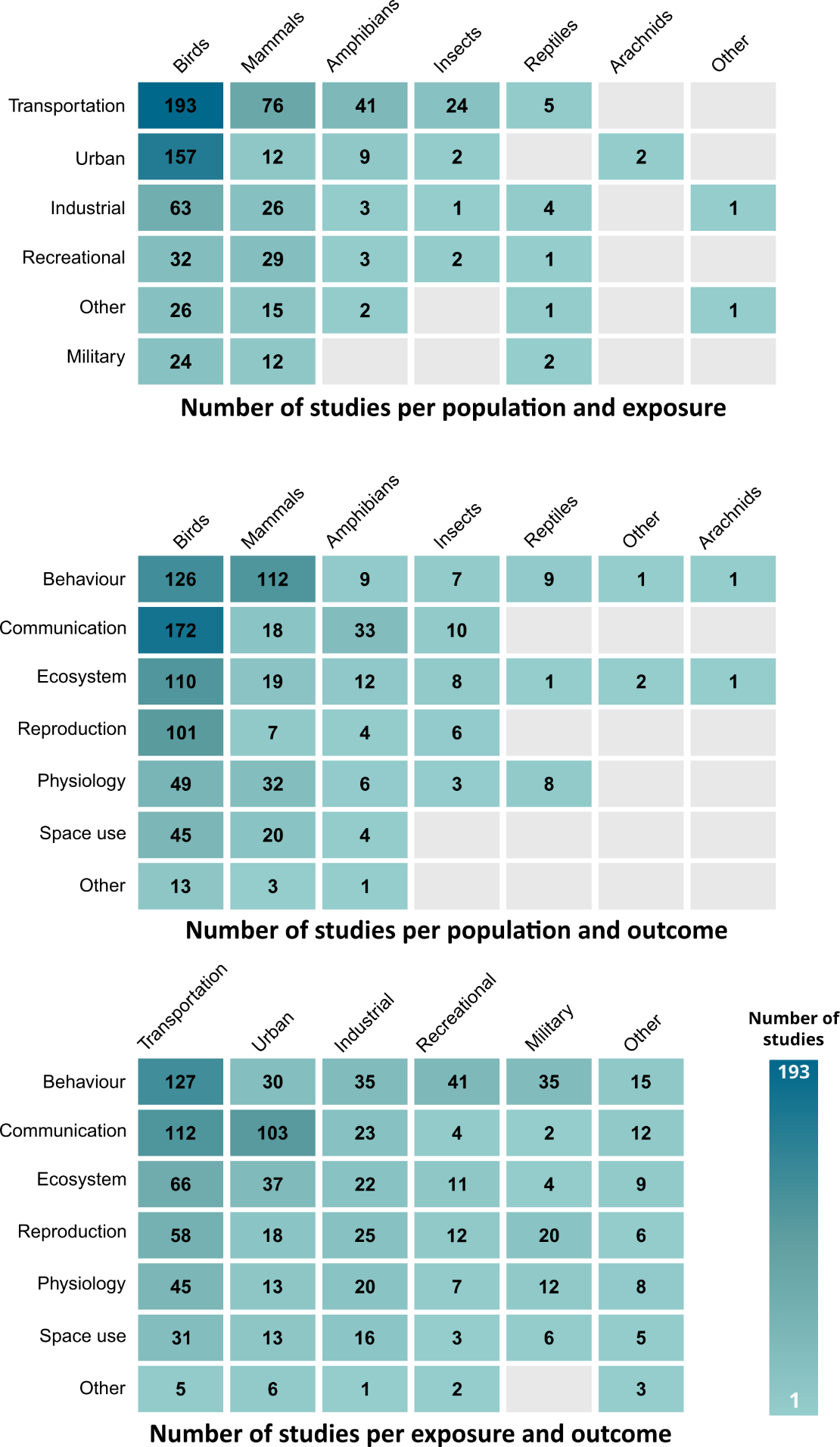


**Number of studies per noise sources and outcomes**

**Additional file 9** Crossed tables detailing the number of studies associated with each pair of key elements of the question (taxonomic groups, outcomes, noise sources).
